# The role of the gut microbiome in the regulation of high‐altitude adaptation

**DOI:** 10.1002/imt2.70104

**Published:** 2026-01-19

**Authors:** Xinyu Zhang, Senlin Zhu, Michael Kreuzer, Shoukun Ji, Wei Wang, Yanliang Bi, Shengli Li

**Affiliations:** ^1^ State Key Laboratory of Animal Nutrition and Feeding, College of Animal Science and Technology, China Agricultural University Beijing China; ^2^ ETH Zurich, Animal Nutrition, Institute of Agricultural Sciences Zurich Switzerland; ^3^ Laboratory of Dairy Cow Genetic Improvement and Milk Quality Research of Zhejiang Province, College of Animal Sciences, Zhejiang University Hangzhou China; ^4^ College of Animal Science and Technology, Hebei Agricultural University Baoding China; ^5^ Key Laboratory of Feed Biotechnology of the Ministry of Agriculture and Rural Affairs, Institute of Feed Research of Chinese Academy of Agricultural Sciences Beijing China

**Keywords:** high altitude adaptation, rumen epithelial cell, rumen microbiome, yak

## Abstract

Hypoxia and cold temperatures are major limiting factors for animals reared at high altitudes. Previous adaptation studies have primarily focused on genetic and genomic aspects, while the mechanisms by which the gut microbiome contributes to this adaptation are still not fully understood. We used ruminants as both naturally adapted (yaks) and non‐adapted (Holstein cows) models to investigate the role of gut microbiome in high‐altitude adaptation by applying multi‐omics approaches. First, 20 yaks and 20 Holstein cows that had been reared at approximately 4000 m altitude since birth were fed the same diet for 44 days prior to sampling to eliminate the short‐term effects of nutrition and altitude adaptation. The yak rumen microbiome showed significant enrichment in carbon metabolism, particularly central carbon metabolism pathways, such as glycolysis/gluconeogenesis, pyruvate metabolism, and the pentose phosphate pathway, whereas that of Holstein cows was enriched in starch, sucrose, pentose, and glucuronate interconversions. Compared with those of Holstein cows kept at high altitudes for their entire life, the yak rumen epithelial cells, as determined by single‐nucleus RNA sequencing, exhibited higher elevated scores for ketone body biosynthesis and fatty acid beta‐oxidation. Second, mixed rumen fluid was transplanted from 10 yaks to 10 Holstein cows. Holstein cows then showed better milk production performance. A progressive decline in carbon metabolism activity from 6 h to 7 and 28 days post‐transplantation was verified. In conclusion, the rumen microbiome and host epithelial function appear to support high‐altitude adaptation by improving the energy supply of the host.

## Highlights

- Multi‐omics integration revealed species‐specific rumen energy metabolic strategies in yaks and Holstein cows under extreme high altitude environment.
- Compared with those of Holstein cows reared at high altitudes their entire lives, yak rumen epithelial cells exhibited enhanced ketone body synthesis and fatty acid beta‐oxidation.
- Yak rumen fluid transplantation confirmed enhanced microbial central carbon metabolism and resulted in improved milk production in Holstein cows.

## Graphical abstract

This study is the first to elucidate mechanisms of high‐altitude adaptation from the perspective of the rumen ecosystem by using indigenous yaks and Holstein cows that have lived at high altitude since birth as comparative models. Through a systematic comparison of their rumen ecology using multi‐omics approaches—including rumen metagenomics, metatranscriptomics, and single‐nucleus transcriptomics—we identified distinct adaptive strategies employed by the two species under extreme plateau environments. Specifically, the rumen microbiota of yaks was enriched in central carbon metabolism pathways, whereas the microbiota of Holstein cows was enriched in pathways related to the degradation of specific complex carbohydrates. Concurrently, yak rumen epithelial cells exhibited increased expression of genes associated with ketone body metabolism, along with higher pathway activity scores for ketone body biosynthesis and fatty acid beta‐oxidation. Moreover, we introduced rumen fluid transplantation from yaks for the first time and observed that, under the conditions of this study, yak microbiota transplantation increased milk yield in Holstein cows. After transplantation, the carbon metabolism of the rumen microbiota gradually declined, further confirming the key role of the rumen microbiota in regulating host energy supply in the context of yak high‐altitude adaptation. Overall, this study deciphers the mechanisms by which gastrointestinal microbiota contributes to high‐altitude adaptation in ruminants and provides a new perspective for addressing challenges related to adaptation to high‐altitude environments.

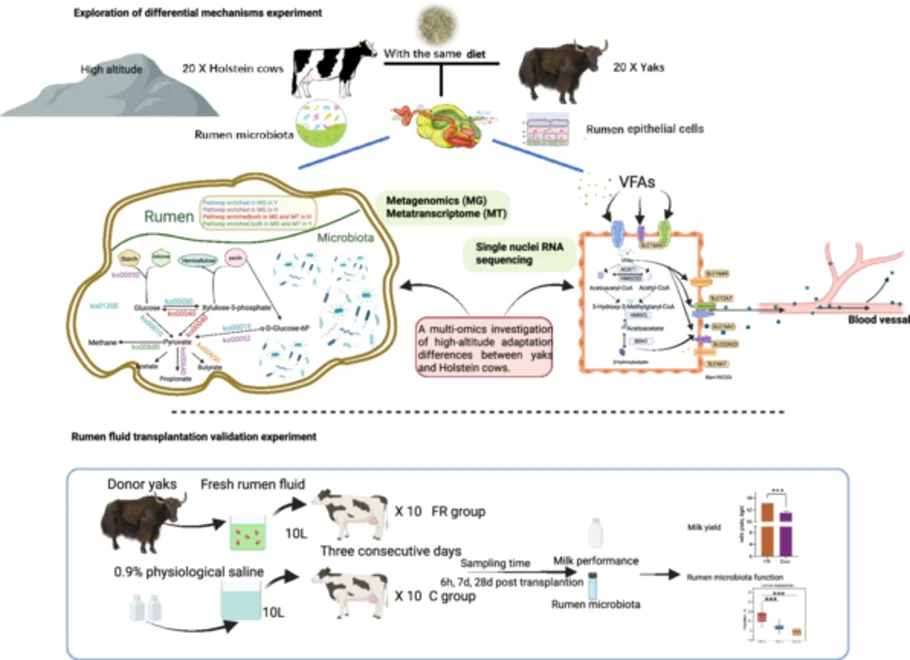

## INTRODUCTION

Extreme conditions at high altitudes, including low oxygen pressure, intense ultraviolet radiation, and low temperatures, lead to hypoxia and increased metabolic demands, posing significant physiological challenges to mammalian survival. Ruminants convert human‐inedible, low‐value plant biomass into nutrient‐rich milk and meat [1, 2]. This process relies on the ruminal microbiome, which ferments plant cell wall carbohydrates into volatile fatty acids (VFAs) through biochemical pathways [3]. These VFAs are absorbed and metabolized by the stratified squamous epithelium of the rumen, a process facilitated by diverse cell types, and are a major energy source for ruminant metabolism. Alterations in the rumen microbiome and epithelial cell function may influence energy supply and, consequently, affect animal performance, including growth and milk yield. Accordingly, Qiao et al. [4] reported that the production efficiency of dairy cows in high‐altitude regions is lower than that in low‐altitude regions. Therefore, given that the specific high‐altitude conditions impose increased metabolic demands, a stable and sustained energy supply is critical for adaptation to high altitudes. Maintaining gut microbiome homeostasis is thus essential for ensuring adequate energy supply.

Among ruminants, the yak (*Bos grunniens*), which was domesticated approximately 7300 years ago, is an excellent model for studying the mechanisms of high‐altitude adaptation developed through long‐term evolution and yak breeding. Various adaptive mechanisms have been identified in yaks compared with ruminants reared in lowlands. Yaks can prosper on local vegetation, which is dominated by plants poor in nutrients and rich in indigestible fiber [5], because they have superior fiber digestion and dietary nitrogen utilization capabilities [6]. Furthermore, there are various modes of adaptation in molecular phylogeny, morphological specificity, and physiological function, including variations in the genome [7, 8] and DNA methylation [9], which differ from those of lowland ruminant species. The unique genomic features and tightly regulated gene expression of yak underline their physiological resilience to hypoxia. However, research on the differences in the gastrointestinal microbiome and their role in the environmental adaptation of yaks is lacking. Although evidence remains limited, Zhang et al. [10] reported that, compared with cattle living at low altitudes, the yak rumen microbiome exhibits a greater abundance of VFA fermentation pathways, and the yak rumen epithelium is more efficient in VFA absorption. However, these findings may be confounded by dietary and altitudinal factors.

This study aimed to systematically investigate the differences in high‐altitude adaptation strategies between indigenous yaks and a closely related species, cattle (*Bos taurus*), from the perspective of rumen ecology. We selected Holstein cows for this purpose because this breed is of pure lowland origin and has been bred for extremely high performance. To ensure comparability, both animal types used in this study were reared at high altitudes since birth. We employed a multi‐omics approach, including metagenomics and meta‐transcriptomics; further, we applied single‐nuclei RNA sequencing (snRNA‐seq) to rumen epithelial cells as an indirect means of assessing cell‐type‐specific functions. This approach enabled us to verify the contribution of ruminal microbial function to adaptability. However, to the best of our knowledge, no detailed description of this mechanism is currently available. We hypothesized that the two animal species would exhibit distinct high‐altitude adaptation strategies, specifically reflected in the rumen system, as follows: (1) differences in the structure and function of the rumen microbiome to mediate host energy supply, influencing high‐altitude adaptability, and (2) in response to differences in rumen‐derived energy supply, the absorption and metabolic functions of the rumen epithelial cells differ. Accordingly, we compared the structural and functional differences between the yak and Holstein cow microbiomes, along with the metabolic differences in rumen epithelium. Subsequently, we transplanted the rumen microbiome of yaks into Holstein cows to verify whether the transfer could improve the host's adaptation and performance. The resulting data provide crucial insights into the rumen microbiome and the mechanism of rumen epithelial cellular metabolism underlying the adaptive evolution of the model animal (yak), and important insights into the mechanisms by which yaks adapt to their high‐altitude environment through rumen microbiome and epithelial cell energy metabolism. These adaptations may extend to the gut microbiomes of other mammals living permanently at high altitudes, and complement previous findings from hypoxia‐focused studies in both ruminants [11] and monogastrics [12], including humans [13].

## RESULTS

### Differences in indicators of rumen fermentation between yaks and Holstein cows

Indicators of the intensity and nature of rumen fermentation showed that the rumen fluid of yaks, a high‐altitude‐adapted species, had a higher concentration of total VFAs than that of Holstein cows (*p* < 0.01) and exhibited a trend toward a lower acetate/propionate (A/P) ratio (3.5:1 vs. 3.8:1, Table S1), whereas no significant difference was observed in serum glucose levels between the two species [6].

### Differences in microbiome characteristics between yaks and Holstein cows as determined by metagenomic profiling

Metagenomic sequencing performed with the rumen fluid samples collected from all 20 Holstein cows and 20 yaks yielded 701 Gb of data, with 17.5 ± 0.3 Gb (mean ± standard error of mean [SEM]) per sample, and 4.6 × 10^9^ reads were generated, with 1.2 ± 0.1 × 10^8^ reads per sample (Table S2). After quality control by removing low‐quality sequences (setting the base pairs (bp) ≥ 20) and host genes, a total of 3.6 × 10^9^ clean reads were retained, with 9.0 ± 1.1 × 10^7^ reads per sample. After *de novo* assembly in MEGAHIT V1.1.2 (setting the minimal length contig ≥ 300 bp), a total of 6.0 × 10^7^ contigs were generated (with an N50 length of 613 ± 5 bp), with 1.5 ± 0.2 × 10^6^ contigs per sample.

The rumen metagenome comprised 94.44% bacteria (1.4 × 10^9^ sequences), 3.95% archaea (5.7 × 10^7^ sequences), 1.07% viruses (1.5 × 10^7^ sequences), 0.53% eukaryotes (7.6 × 10^6^ sequences), and 0.02% unclassified domains (2.3 × 10^5^ sequences; Table S3). These data were subjected to intensive analyses and used for statistical comparisons between yaks and Holstein cows. The comparisons focused on bacteria and archaea, the two dominant microbial domains, which together accounted for >98.4% of the total sequencing reads. Yaks showed significantly higher Chao1 and Simpson indices for community, but a lower Shannon index than Holstein cows (*p *< 0.001, Figure S1A). Alpha diversity also differed between the groups for both archaea (Figure S1B) and bacteria (Figure S1C). Comparison of the rumen microbial domains also revealed differences between yaks and Holstein cows in the relative abundance of archaea and bacteria (adjusted *p *< 0.001; Figure 1A). Principal coordinate analysis (PCoA) illustrated the separation in the archaeal (Figure 1B) and bacterial (Figure 1C) species between yaks and Holstein cows, which was further supported by PERMANOVA (adjusted *p *= 0.001).

**Figure 1:**
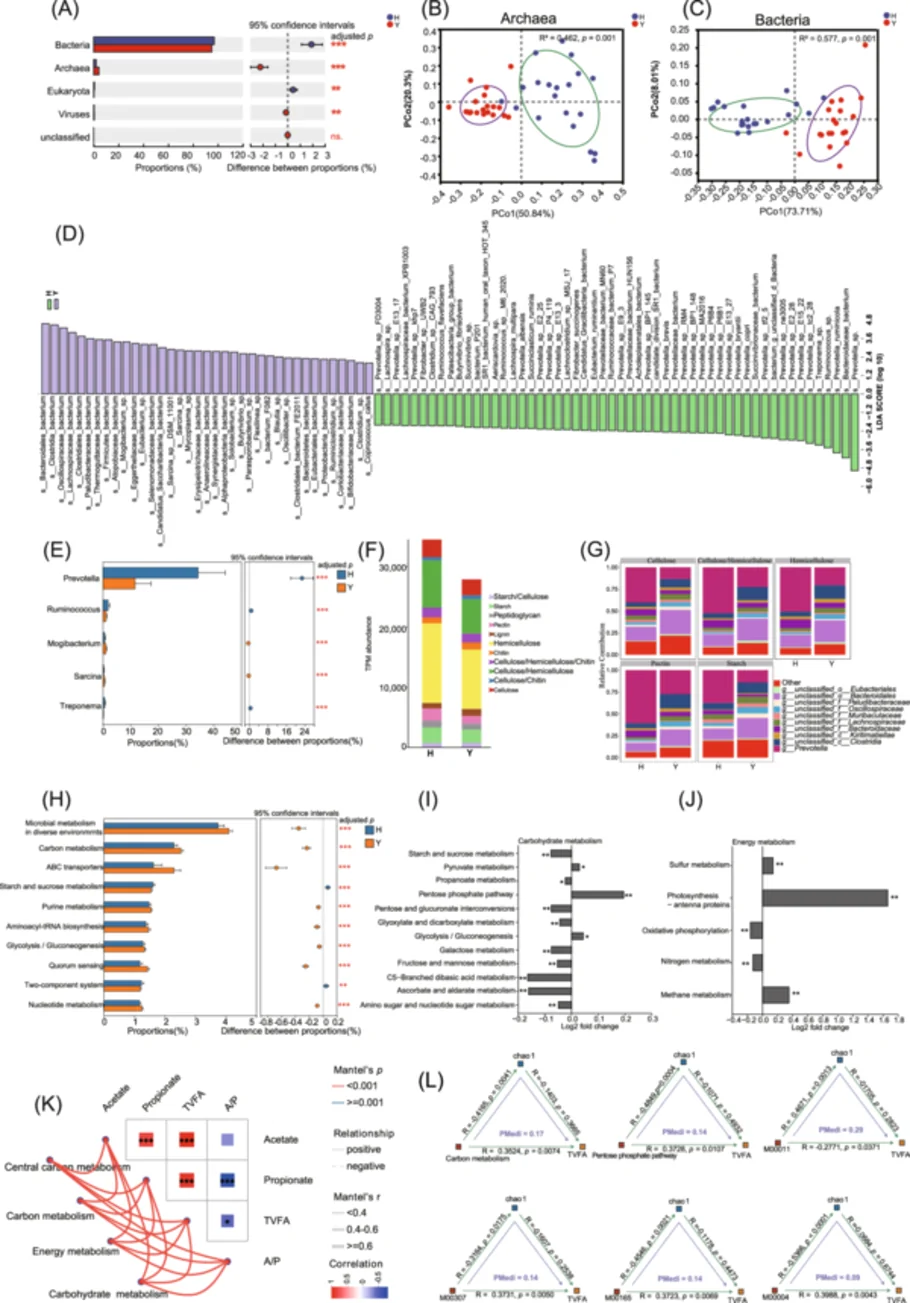
Differences between Holstein cows (H) and yaks (Y) in microbial taxonomic composition and functional potential in the rumen fluid, as determined by metagenomic analysis (*n* = 20 per group; Experiment 1). (A) Differences in microbial composition (domain level) between Holstein cows and yaks, with Wilcoxon rank sum test being used to test for significant differences. Differences in rumen archaea (B) and bacteria (C) between Holstein cows and yaks at species level as visualized by using PCoA. (D) Differences in common bacterial species composition between Holstein cows and yaks determined by Linear discriminant analysis Effect Size. (E) Top five differential genera identified based on the Wilcoxon rank‐sum test. (F) Stacked bar chart of carbohydrate degradation enzyme function. (G) Bar chart of CAZyme functional contribution analysis. (H) Top 10 differential Kyoto Encyclopedia of Genes and Genomes (KEGG) level 3 pathways identified based on the Wilcoxon rank‐sum test. KEGG pathways were sorted for carbohydrate metabolism (I) and energy metabolism (J) identified in the metagenomes. (K) Mantel test analysis between VFA concentration and carbon metabolism, central carbon metabolism, carbohydrate metabolism, and energy metabolism in yaks and Holstein cows. (L) Established mediating effect models. The figures above the arrowed lines indicate the regression coefficient. The asterisk indicates the significant regression relation. The numbers in the triangle indicate the mediation effects. *adjusted *p* < 0.05; **adjusted *p* < 0.01; ***adjusted *p* < 0.001.

We distinguished microbial species that were unique to yaks, unique to Holstein cows, and common to both (microbial species were defined as “unique to yaks” if they were present in over 50% of yak samples but in fewer than 50% of Holstein cow samples; similarly, species were defined as unique to Holstein cows or common to yaks and Holstein cows). Accordingly, 2299 species were unique to yaks, 2511 were unique to Holstein cows, and 10,902 were shared (Figure S2A). The most dominant bacterial phyla unique to yaks were *Firmicutes* (*Bacillota*, 35.1%), *Proteobacteria* (22.1%), *Bacteroidota* (15.1%), and *Actinobacteria* (12.5%); in comparison, those unique to Holstein cows were *Proteobacteria* (27.7%), *Bacteroidota* (24.7%), *Firmicutes* (*Bacillota*, 19.9%), and *Actinobacteria* (5.7%). The three most dominant archaeal phyla unique to yaks were *Euryarchaeota* (80.6%), *Candidatus Thermoplasmatota* (4.0%), and *Candidatus Micrarchaeota* (2.4%), and those unique to Holstein cows were *Euryarchaeota* (36.1%), *Thaumarchaeota* (14.2%), and *Crenarchaeota* (9.5%, Figure S2A).

Regarding archaeal species shared between yaks and Holstein cows, 35 were more abundant in yaks, whereas 20 showed a higher relative abundance in Holstein cows (all linear discriminant analyses [LDA] > 2, *p *< 0.05; Figure S2B). At the bacterial species level, 37 were more abundant in yaks (LDA > 2, *p *< 0.05), whereas 54, including 25 belonging to *Prevotella*, were more abundant in Holstein cows (LDA > 2, *p *< 0.05; Figure 1D). Based on these findings, we conducted a Wilcoxon rank‐sum test at the genus level to analyze the differential abundance of the top five shared bacterial genera between the two groups (Figure 1E). Among the top five differentially abundant genera, *Prevotella*, *Ruminococcus*, and *Treponema* were significantly enriched in the Holstein cow group (adjusted *p *< 0.001), whereas *Mogibacterium* and *Sarcina* were significantly enriched in yaks (adjusted *p *< 0.001).

The correlations between the abundance of individual rumen microbial species and end products of ruminal fermentation, including the concentrations of acetic acid, propionic acid, butyric acid, valeric acid, total VFAs, A/P ratio, ammonia nitrogen (NH_3_‐N), and rumen pH, were determined and are presented in heatmaps. The abundances of a few archaeal species unique to yaks and Holstein cows were significantly correlated with fermentation traits (Figure S2C,D), but more so for unique bacterial species (Figure S2E,F) based on Spearman's rank correlation analysis. Correlations with the relative abundance of microbial species common to animal species were observed for various fermentation traits (Figure S2G–J). Twenty‐one bacterial species belonging to *Prevotella* were shared in Holstein cows, and were negatively correlated with ruminal pH (Figure S2I). A detailed description of the results of the correlation analysis is provided in the Supplementary Material.

Gene expression levels related to rumen microbiome functions were determined from metagenomic data using genes encoding carbohydrate‐active enzymes (CAZymes) and Kyoto Encyclopedia of Genes and Genomes (KEGG) profiles; 7 classes of CAZymes were identified, belonging to 25 auxiliary activities (AAs), 80 carbohydrate‐binding modules (CBMs), 16 carbohydrate esterases (CEs), 298 glycoside hydrolases (GHs), 111 glycosyltransferases (GTs), 102 polysaccharide lyases (PLs), and 1 cellulosome module (SLH). The CAZyme composition at the class and family levels concerning gene expression levels is shown in Figure S3A and S3B for Holstein cows and yaks, respectively. In Holstein cows, genes encoding GHs (55.7%) were dominant, followed by those encoding GTs (21.1%), CEs (12.9%), CBMs (5.4%), PLs (2.9%), and AAs (1.9%). Similarly, in yaks, genes encoding GHs (51.8%) were dominant, followed by those encoding GTs (23.1%), CEs (14.3%), CBMs (5.2%), AAs (2.9%), and PLs (2.4%). Genes encoding GHs, CBMs, and PLs were enriched in Holstein cows (adjusted *p <*0.05, Figure S3C), whereas those encoding GTs, CEs, AAs, and SLHs were enriched in yaks (adjusted *p <* 0.001, Figure S3C). Among the GTs involved in carbohydrate synthesis, genes encoding GT2_Giycos_transf_2 and GT4 were enriched in yaks (adjusted *p <* 0.05, Figure S3D). Genes encoding GHs, GH2, GH97, and GH31, which are involved in complex carbohydrate degradation, were enriched in Holstein cows (adjusted *p *< 0.001), whereas genes encoding GH3 and GH94 were enriched in yaks (adjusted *p *< 0.001). Among the genes encoding CEs, those encoding CE1 and CE10 were enriched in yaks (adjusted *p* < 0.001), as were those encoding AA6 were enriched in yaks as well (adjusted *p *< 0.001). In terms of gene abundance, yaks had lower levels of carbohydrate‐degrading enzymes than Holstein cows did (Figure 1F). Specifically, yaks exhibited a significantly higher transcript per million (TPM) mapped reads abundance for lignin and chitin (adjusted *p* < 0.05, Figure S3E), whereas Holstein cows harbored significantly more genes targeting peptidoglycan, pectin, starch, cellulose, and hemicellulose (adjusted *p* < 0.001, Figure S3E). Based on the correspondence between the abundance of microbial species and enrichment of gene‐encoding functions, an association analysis was performed between the top 10 species at the genus level and the relative abundance of functions to identify the functional contributions of specific species (Figure 1G). *Prevotella* exhibited marked functional variation between yaks and Holstein cows.

For KEGG profiles, 454 endogenous third‐level pathways were considered as rumen microbial metabolic pathways (Table S4), which belonged to 6 first‐level categories: metabolism (49.4%), genetic information processing (19.0%), environmental information processing (11.7%), cellular processes (8.8%), human diseases (6.8%), and organismal systems (4.3%; Table S5). In total, 395 third‐level pathways were compared for significant differences between yaks and Holstein cows (Table S4). The 10 most significantly different metabolic pathways between the groups revealed clear functional differences in rumen microbiome composition (Figure 1H). Carbon metabolism ranked second among the top differential pathways in this study. In parallel, we further selected the pathways specifically associated with nutrient digestion and metabolism, including those involved in carbohydrate metabolism (Figure 1I), energy metabolism (Figure 1J), and amino acid (Figure S3F) and lipid metabolism (Figure S3G), primarily focusing on those that were significant at least at adjusted *p* < 0.05. Regarding carbohydrate metabolism, gene expression in three pathways, including pyruvate metabolism, pentose phosphate, and glycolysis/gluconeogenesis, was enriched in yaks compared with Holstein cows, whereas the opposite was observed for nine pathways (starch and sucrose metabolism, pentose and glucuronate interconversions, galactose metabolism, and fructose and mannose metabolism, among others, Figure 1I). Regarding energy metabolism, methane and sulfur metabolism were enriched in yaks, whereas oxidative phosphorylation and nitrogen metabolism were enriched in Holstein cows (Figure 1J).

Carbon, central carbon, carbohydrate, and energy metabolism were positively correlated with VFA concentrations (including acetate, propionate, total VFAs, and A/P; Figure 1K) based on Mantel analysis. Simultaneously, the mediating effect model showed that independent variables, including carbon metabolism, the pentose phosphate pathway, *Prevotella*, *Ruminococcus*, *Mogibacterium*, and the module involved in central carbon metabolism, were correlated with mediating variables, including Chao1 indices, which eventually correlated with A/P (Figures 1L and S4). According to the mediating effect model conditions, eleven mediating effects were established (carbon metabolism→Chao1→TVFA; pentose phosphate pathway→Chao1→TVFA; M00011→Chao1→TVFA; M00307→Chao1→TVFA; M00165→Chao1→TVFA; M00004→Chao1→TVFA; M00007→Chao1→TVFA; *Prevotella*→Chao1→TVFA; *Ruminococcus*→Chao1→TVFA; *Treponema*→Chao1→TVFA; *Mogibacterium*→Chao1→TVFA).

### Differences between yaks and Holstein cows in microbiome characteristics as determined by meta‐transcriptomic profiling

The meta‐transcriptome profiling revealed the active expression of genes and their metabolic functions. This type of sequencing, conducted in rumen fluid samples of a subset of 10 Holstein cows and 10 yaks, generated 358 Gb of data (17.9 ± 0.4 Gb (mean ± SEM) per sample). A total of 156 Gb of data remained after quality control, wherein host RNA reads were filtered out and rRNA data were deleted as the latter did not reflect gene expression levels or the functional status of the microbial community (Table S6). After *de novo* assembly, an average of 1.6 ± 0.11 × 10^5^ sequences were obtained for each sample.

Applying meta‐transcriptomics, there were no significant differences between the two groups in the Chao 1, Shannon, and Simpson indices of all microbial species (*p* > 0.05, Figure S5A), whereas the Shannon and Simpson indices of archaea (*p *< 0.01, Figure S5B) and the Chao1 and Shannon indices of bacteria (*p *< 0.05, Figure S5C) were lower in yaks than in Holstein cows. A comparison of the rumen microbial structure between yaks and Holstein cows revealed no significant differences in the proportions of archaea and bacteria (adjusted *p* > 0.05, Figure 2A). PCoA performed with archaeal (PERMANOVA: *p *= 0.002, Figure 2B) and bacterial (PERMANOVA: *p *= 0.001, Figure 2C) data showed clear separation between the yaks and Holstein cows.

**Figure 2:**
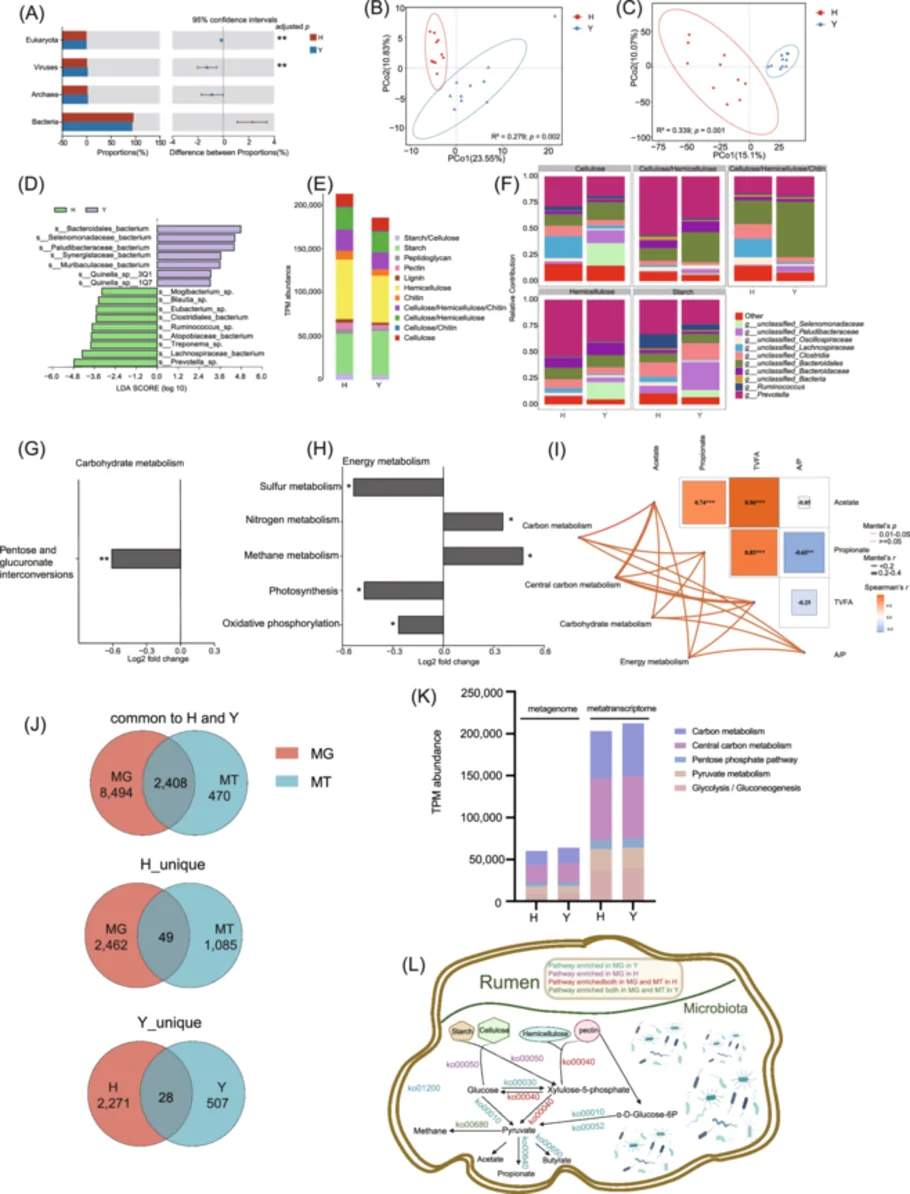
Differences in rumen microbial composition and functional gene expression between Holstein cows (H) and yaks (Y), as determined by metatranscriptomic analysis of rumen fluid samples (*n* = 10 per group; Experiment 1). (A) Differences in rumen microbiome (domain level) between Holstein cows and yaks, with Wilcoxon rank sum test being used to determine significant differences. Differences in rumen archaea (B) and bacteria (C) between Holstein cows and yaks at species level as visualized by using PCoA. (D) Differences in common bacterial species composition between Holstein cows and yaks determined by Linear discriminant analysis Effect Size. (E) Stacked bar chart of carbohydrate degradation enzyme function. (F) Bar chart of genera and functional contribution analysis. Carbohydrate metabolism (G) and energy metabolism (H) based on KEGG pathways identified in the metatranscriptome. (I) Mantel test analysis between VFA concentration and carbon metabolism, central carbon metabolism, carbohydrate metabolism, and energy metabolism in yaks and Holstein cows. (J) Venn diagram of microbial species identified between the metagenome (MG) and metatranscriptome (MT) levels. (K) Comparison of transcripts per million (TPM) mapped reads abundance of key metabolic pathways between the metagenome and metatranscriptome. (L) Schematic summary for the upregulated and downregulated key metabolism pathways in the rumens of two groups using metagenomics and metatranscriptome. *adjusted *p* < 0.05; **adjusted *p* < 0.01; ***adjusted *p* < 0.001.

Downstream comparative analysis revealed 536 microbial species unique to yaks, 1134 unique to Holstein cows, and 2879 common to yaks and Holstein cows (Figure S5D). The top five dominant yak‐specific bacterial phyla were *Bacillota* (73.6%), *Bacteroidetes* (10.5%), *Pseudomonadota* (4.7%), *Actinomycetota* (4.5%), and *Thermodesulfobacteria* (1.6%). The top five dominant Holstein cow‐specific bacterial phyla were *Bacillota* (48.8%), *Actinomycetota* (17.6%), *Pseudomonadota* (11.0%), *Bacteroidota* (8.7%), and *Melainabacteria* (1.7%). The top three yak‐specific archaeal phyla were *Euryarchaeota* (90.4%), *Nitrososphaerota* (6.2%), and *Thermoplasmatota* (3.4%), whereas the top three Holstein cow‐specific archaeal phyla were *Euryarchaeota* (72.9%), *Thaumarchaeota* (21.2%), and *Huberarchaeota* (2.3%). Based on the Linear discriminant analysis Effect Size comparison, the abundance of 28 species of archaea common to yaks and Holstein cows was higher in yaks than in Holstein cows, whereas the abundances of 16 species in Holstein cows were increased compared with yaks (LDA > 2, *p *< 0.05, Figure S5E). For the shared bacteria, nine species and seven species showed higher abundances in Holstein cows; among them, Prevotella sp. was significantly enriched in Holstein cows (LDA > 2, *p* < 0.05; Figure 2D). At the genus level, *Prevotella*, a dominant taxon, showed a trend towards a higher abundance in Holstein cows (adjusted *p* = 0.062, Table S7). Consistent with the metagenomic results, yaks exhibited lower levels of carbohydrate‐degrading enzymes than Holstein cows (Figure 2E), specifically, yaks exhibited a higher TPM mapped reads abundance for starch; however, this difference was not statistically significant (adjusted *p* > 0.05, Figure S5F), whereas Holstein cows harbored significantly more genes targeting peptidoglycan, pectin (adjusted *p* < 0.001, Figure S5F). And *Prevotella* exhibited marked functional variation between yaks and Holstein cows (Figure 2F).

In the rumen meta‐transcriptomic analysis, 412 KEGG level‐3 metabolic pathways were identified, of which 168 pathways differed between Holstein cows and yaks (adjusted *p *< 0.05, Table S8). In carbohydrate metabolism, only pentose and glucuronate interconversions were enriched in Holstein cows compared with yaks (adjusted *p *< 0.05, Figure 2G). Regarding energy metabolism, three pathways were enriched in Holstein cows and two in yaks (adjusted *p *< 0.05, Figure 2H). Regarding amino acid metabolism pathways, two each were enriched in yaks and Holstein cows (adjusted *p *< 0.05, Figure S5G). Regarding lipid metabolism, seven pathways were enriched in Holstein cows and one in yaks (adjusted *p *< 0.05, Figure S5H). Meta‐transcriptomic analysis also revealed that carbon, central carbon, carbohydrate, and energy metabolism were significantly positively correlated with VFA concentrations (including acetate, propionate, total VFAs, and A/P ratio; Figure 2I), consistent with the metagenomic findings.

### Comparison of DNA‐ and RNA‐based determinations of microbial composition and profiling of functions between yaks and Holstein cows

We detected microbial composition and functions that were either shared or unique between the two omics data sets within each respective niche. Specifically, the metagenome analysis identified greater numbers of microbial species unique to yaks, unique to Holstein cows, and common to yaks and Holstein cows compared with the meta‐transcriptome analysis (Figure 2J). The microbiome data (including both metagenomic and meta‐transcriptomic analyses) further showed that yaks favored central carbon and carbon metabolism based at both the DNA and RNA levels (Figure 2K), but Holstein cows exhibited a greater diversity in sugar‐specific degradation pathways than yaks (Figure 2L). Specifically, Holstein cows were significantly enriched in sugar‐specific degradation pathways, such as pentose and glucuronate interconversions, starch and sucrose metabolism, galactose metabolism, and amino sugar and nucleotide sugar metabolism (Figure 2L). A more detailed metabolic pathway diagram, including core modules and key enzymes, along with their expression profiles in both the metagenome and meta‐transcriptome, is presented in Figure 3.

**Figure 3:**
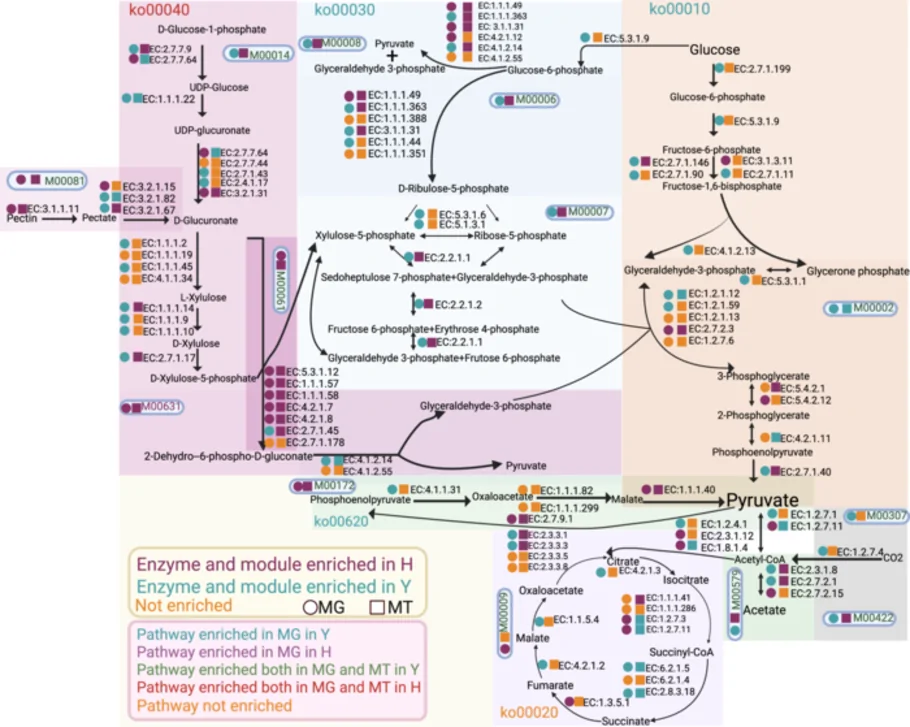
Modules and enzymes of central carbon metabolism and key carbohydrate and energy metabolism pathway diagram based on results of metagenomics and metatranscriptome analysis. MG, metagenomics; MT, metatranscriptomics.

### Differences between yaks and Holstein cows in rumen epithelial characteristics as determined by single‐nucleus RNA sequencing (snRNA‐seq)

The analysis revealed 33 and 27 clusters from 45,682 and 34,280 high‐quality individual epithelial cells obtained from the yaks and Holstein cows, respectively. The snRNA‐seq analysis overlap indicated high data fidelity and reproducibility of the rumen epithelial cell landscape, both across individuals (Figure S6A) and within cell clustering (Figure S6B). We divided all harvested cells into different cell types (Figure 4A,B) based on their marker genes [14] (Figure S6C and S6D for Holstein cows and yaks, respectively). We further extracted rumen epithelial cells and reclustered them to obtain 13 and 14 subtypes of rumen epithelial cells in Holstein cows (Figure 4C) and yaks (Figure 4D), respectively, based on marker genes (Figure 4E,F). The reclustering of other cell subtypes is shown in Figures S6E and S6F for Holstein cows and yaks, respectively. Figure S7A shows the similarities between Holstein cow and yak rumen epithelial cell subtypes using Metaneighbor.

**Figure 4:**
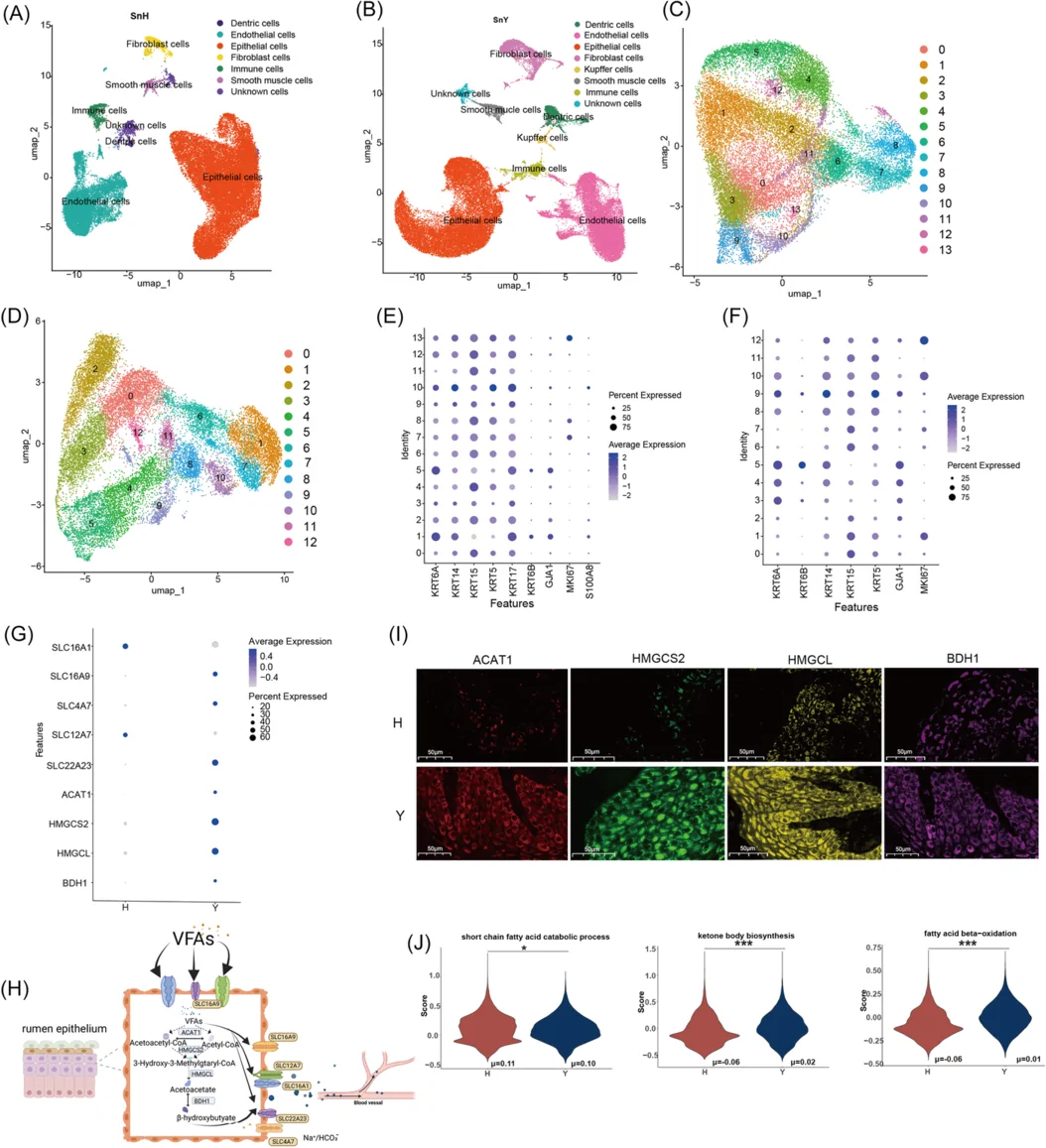
Differences between the Holstein cows (H) and yaks (Y) as illustrated by a rumen epithelial single‐cell map (*n* = 3 per group; Experiment 1). Uniform manifold approximation and projection (UMAP) maps of cells from Holstein cows (A) and yaks (B) rumen epithelial tissue samples, with each cell color indicating the associated cell types. (C, D) UMAP map of rumen epithelial cell subtypes of Holstein cows and yaks. Marker gene expression in single‐cell subtypes of Holstein cows (E) and yaks (F). (G) Differences between Holstein cows and yaks in the cell‐type‐enriched expression of solute carrier (SLC) and ketone body biosynthesis metabolism genes. (H) Schematic diagram of VFAs and ketone body biosynthesis and transport. (I) Immunostaining of ketone body metabolism enzymes: ACAT1, HMGCS2, HMGCL, and BDH1. (J) Differences between Holstein cows and yaks in the score of pathways of short‐chain fatty acid catabolic process, fatty acid beta‐oxidation, and ketone body biosynthesis metabolism. adjusted *p* < 0.05; **adjusted *p* < 0.01; ***adjusted *p* < 0.001.

Figure S7B shows the pathways associated with the top 15 genes in yaks and Holstein cows, as identified by Gene Ontology (GO) functional enrichment analysis. Energy metabolism‐related pathways, including the carbohydrate‐derivative biosynthetic, organic acid metabolic, and carboxylic acid metabolic processes, were enriched in Holstein cows. Based on these findings, we focused on the expression of genes involved in energy‐metabolism‐related pathways, including volatile fatty acid transport and ketone‐body metabolism. Among the genes related to VFA transport, *SLC16A9*, *SLC4A7*, and *SLC22A23* were enriched in yaks, whereas *SLC16A1* and *SLC12A7* were enriched in Holstein cows (Figure 4G). Among the pathways related to ketone body metabolism, *ACAT1*, *HMGCS2*, *HMGCL*, and *BDH1* were enriched in yaks (Figure 4G,H), which was further verified by immunofluorescence staining (Figure 4I). By scoring pathways, those related to ketone body biosynthesis and fatty acid beta‐oxidation were enriched in yaks compared with Holstein cows (*p* < 0.001; Figure 4J), and those related to the VFAs catabolic process were enriched in Holstein cows compared to yaks (*p* < 0.05; Figure 4J). The activity scores of different cell subtypes in Holstein cows and yaks in the catabolic process of VFAs, ketone body biosynthesis, and fatty acid beta‐oxidation are shown in Figure S7C–E.

### Effects of transplanting rumen fluid from yaks to Holstein cows to validate the effects of the rumen microbiome on high‐altitude adaptation

The transplantation of the rumen fluid of the donor yaks had highly significant temporal effects on milk, milk protein, and lactose yields in recipient Holstein cows (*p* < 0.001), whereas no significant differences were observed for either treatment or the interaction between time and treatment (*p* > 0.05, Figure 5A). Specifically, on Day 7 post‐transplantation, milk and lactose yields were higher in the transplant group (FR) than those in the control group (Con; *p* < 0.05). On Day 14 post‐transplantation, milk protein and lactose yields were higher in the FR group than those in the Con group (*p* < 0.05, Figure 5A). In addition, over the entire 28‐day experimental period, milk yield was significantly higher in the FR group than in the Con group (*p* < 0.001; Figure 5B).

**Figure 5:**
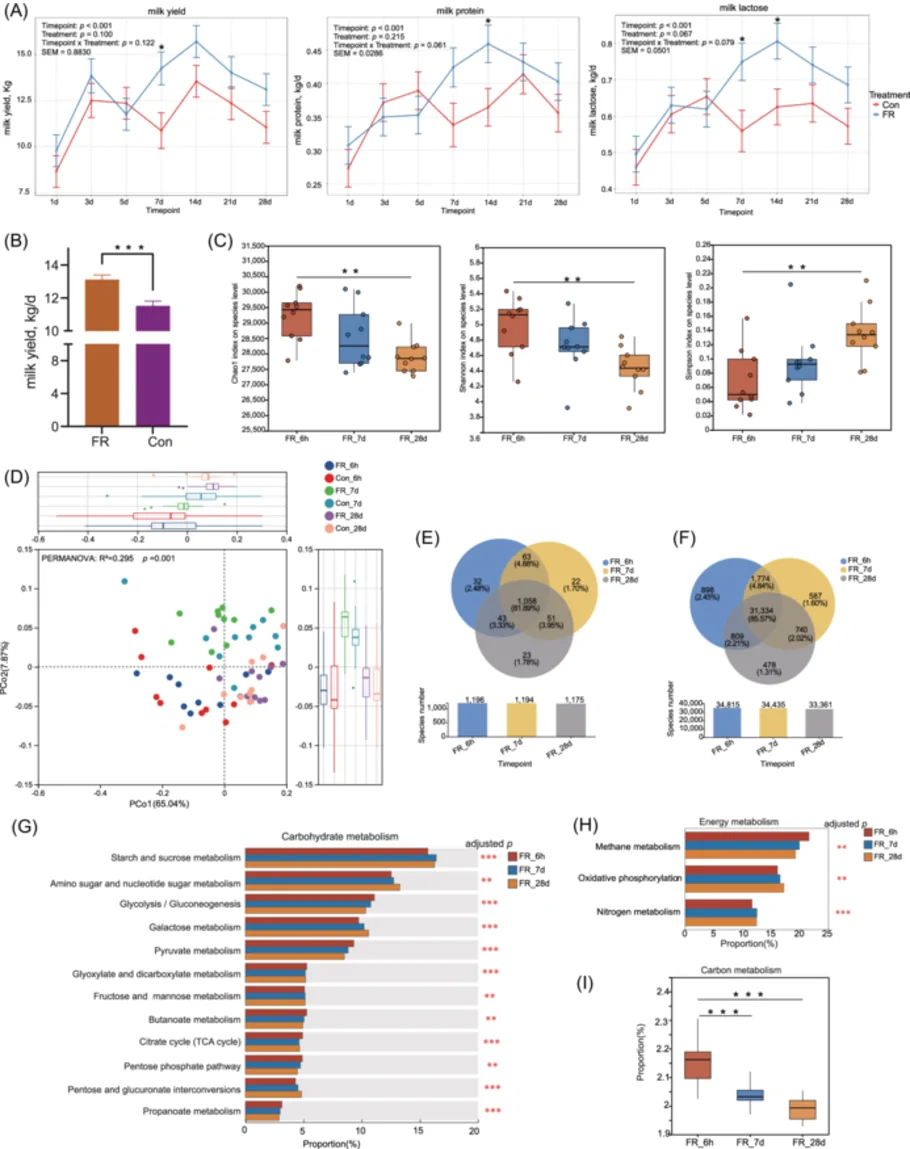
Effect of transplantation of rumen fluid from yaks to Holstein cows on indicators of performance and rumen microbiome function (*n* = 10 per group; FR = Fresh rumen fluid group, Con = Control group; Experiment 2). (A) Effects on milk and milk protein yield, as well as milk lactose yield. (B) Difference in milk yield throughout the 28‐day experimental period as analyzed by a *t*‐test. (C) Evolution of alpha diversity (Chao1, Shannon, and Simpson indices) in the rumen microbiome at 6 h, and on Day 7 and Day 28 post transplantation, assessed using the Wilcoxon rank‐sum test. (D) Differences between different groups at different time points post‐transplantation in rumen microbiome on species level as visualized by using principal coordinate analysis (PCoA). Venn diagrams of archaea (E) and bacteria (F) at species level at 6 h, and on Day 7 and Day 28 post‐transplantation. Evolution of KEGG level 3 pathways with time post‐transplantation sorted by carbohydrate metabolism (G), energy metabolism (H), and carbon metabolism (I). *adjusted *p* < 0.05; **adjusted *p* < 0.01; ***adjusted *p* < 0.001.

The microbial community underwent selection at 6 h, and on Day 7 and Day 28 post transplantation, and the metagenomic sequencing data are presented in Table S9. Specifically, the Chao1 and Shannon diversity indices significantly decreased, whereas the Simpson index significantly increased (*p* < 0.01; Figure 5C). PCoA expression at the species level revealed that the microbiome differed between the two groups at 6 h, and on Day 7 and Day 28 post transplantation (PERMANOVA, *p* = 0.001, Figure 5D). Concurrently, we also observed temporal shifts in the number of species expressed in both archaea (Figure 5E) and bacteria (Figure 5F) across the three time points, indicating dynamic changes in microbial community composition after transplantation. In addition, differences in the relative abundances of the key bacterial genera *Prevotella*, *Ruminococcus*, *Treponema*, and *Mogibacterium* (*p* < 0.05, Figure S8A) were observed at 6 h, and on Day 7 and Day 28 post‐transplantation.

Functional profiling based on KEGG annotation revealed differences in the carbohydrate (Figure 5G), energy (Figure 5H), and carbon (Figure 5I) metabolic pathways between the FR and Con groups. FR exhibited a stronger central carbon metabolism capacity during the early post‐transplantation period, including metabolic pathways such as glycolysis/gluconeogenesis, pentose phosphate, and the citrate cycle (tricarboxylic acid (TCA) cycle), which were highly expressed at 6 h post‐transplantation, followed by a decrease on Day 7 and Day 28 (*p* < 0.01, Figure 5G). In contrast, several pathways related to the degradation of upstream carbon sources, including the metabolism of starch and sucrose, galactose, fructose, and mannose metabolism as well as pentose and glucuronate interconversions, exhibited increases over time post‐transplantation (*p* < 0.001; Figure 5G).

We further summarized the modules of carbon metabolism that exhibited significant differences across metagenomic and meta‐transcriptomic data and that of the rumen microbiome in these two experiments (Figure S8B). This included glycolysis (M00002), pentose phosphate (M00004, M00006, and M00007), and Entner–Doudoroff (M00008), all of which significantly decreased (*p* < 0.05, Figure S8C). These pathways are also integral components of central carbon metabolism. In addition, the methanogenesis modules (M00356 and M00567) and acetate production pathway (M00579) declined (*p* < 0.05, Figure S8C). Conversely, carbon fixation modules, such as the C_4_‐dicarboxylic acid cycle (M00171) and reductive acetyl‐CoA pathway (M00377), first decreased after transplantation and later recovered, whereas the CO₂‐to‐acetyl‐CoA module (M00422) increased transiently (*p* < 0.05, Figure S8C).

## DISCUSSION

### Characteristics of the rumen microbiome and its function in high‐altitude‐adapted yaks compared with that of lowland high‐genetic‐merit Holstein cows

Given its fundamental role in host metabolism, immune regulation, and physiological functions, the gastrointestinal microbiome is also called the “second genome” [15]. The rumen microbiome influences traits such as milk protein production [16], methane formation [17], and feed efficiency [18]. Although diet shapes microbial composition, host genetics also play a crucial role, with microbial species composition being heritable [18]. Different ruminant breeds maintain distinct and stable microbiomes [19], which may result from co‐evolution and long‐term host adaptation. The gut microbiome is currently recognized as a key player in high‐altitude adaptation. This has been observed in recent studies in other ruminants, such as sheep [20] and cervids [11], but also monogastrics, including pigs [12, 21], mice, and even humans [13]. Zhang et al. [20] highlighted how diet–microbiota interactions mediate high‐altitude adaptation in wild herbivores, Li et al. [11] reported microbial genome plasticity in carbohydrate and energy metabolism pathways in cervids on the Tibetan Plateau. Furthermore, Zhao et al. [12] found that members of the *Fibrobacterota* and *Elusimicrobia* are associated with high‐altitude adaptation in Tibetan pigs, and Su et al. [13] identified *Blautia_A* as a key taxon facilitating hypoxic adaptation through anti‐inflammatory and barrier‐protective effects in mouse models and humans using longitudinal sampling and multiple cohorts. Research on the gastrointestinal microbiome, therefore, presents a novel perspective for describing the determinants of enhanced environmental stress tolerance, with implications for well‐being and health in humans.

As fiber degradation is mostly mediated by rumen microbial species (with a low contribution from large intestinal microbial species), the yak rumen microbiome has superior fiber‐degrading capacity. This may explain why yaks exhibited higher energy‐use efficiency than lowland‐adapted cattle. We speculate that, compared with Holstein cows, yaks may utilize other microbial and metabolic strategies developed by long‐term selection to adapt to high altitudes and meet the combined challenges of hypoxia and elevated energy demands. Preliminary findings from yaks and cattle herd surveys demonstrated differences in the rumen microbiome between yaks and cattle; however, these herds were kept at different altitudes [22]. This study is the first to obtain information about the characteristic rumen microbiome of yaks developed by long‐term selection, compared with that of non‐adapted lowland‐bred Holstein cows, by excluding the direct effects of diet and altitude.

The observation that, at the species level, fewer microbial species were identified in yaks than in Holstein cows across both “omics” datasets suggests a more phylogenetically streamlined community structure in the native high‐altitude ruminants. Metagenomic profiling indicated that yaks had a lower Shannon index but higher Chao 1 and Simpson indices than Holstein cows, indicating a microbial community with greater richness, but reduced diversity and evenness, likely dominated by a few highly abundant taxa, along with many low‐abundance members. However, the meta‐transcriptomic data revealed that yaks exhibited lower Chao 1 and Shannon indices and a higher Simpson's index than Holstein cows, although these differences were not statistically significant. This divergence likely reflects the distinction between structural potential and active function; metagenomics captures the total gene pool (including dormant or low‐abundance taxa), whereas meta‐transcriptomics reflects a subset of microbial species that are transcriptionally active under the current physiological conditions. Nevertheless, this pattern implies that compared with Holstein cows, yak microbiomes may exhibit a more specialized, high‐altitude‐adapted community structure with fewer dominant taxa, but less functional redundancy [23]. Studies of the core microbiome have attracted significant attention in recent years, particularly in understanding key microbial functions in specific environments [24]. In this study, we defined common species as the core microbiome and identified microbial species unique to yaks and Holstein cows. The compositional differences in the shared species suggested that these species may have been key taxa contributing to the observed functional differences in the microbiomes. In yaks, the abundance of several *Prevotella* species was significantly lower than that in Holstein cows; these organisms are recognized as key taxa for complex plant polysaccharide degradation. The abundance of the Prevotella species is positively correlated with VFA concentrations, and has been linked to improved host production traits, such as milk yield [25, 26]. Although meta‐transcriptomic profiling detected fewer differentially abundant taxa than metagenomic analysis, a *Prevotella* species was consistently identified among the shared species in yaks and Holstein cows, underscoring its active role under the sampled conditions. We also observed a lower abundance of *Ruminococcus* and *Treponema* in yaks than in Holstein cows. Together, *Ruminococcus* and *Prevotella* function as fibrolytic specialists capable of degrading hemicellulose, proteins, and starch. In contrast, the rumen microbiome of the yaks was enriched in *Mogibacterium* and *Sarcina*, which are associated with protein or amino acid fermentation (*Mogibacterium*) and carbohydrate fermentation (*Sarcina*), respectively. We speculate that, in addition to the well‐established genetic factors, functional differences in the rumen microbiome between yaks and Holstein cows influence the host's energy supply.

Rumen microbial species, equipped with enzymes capable of degrading cellulose, xylan (hemicellulose), and pectin, initiate the breakdown of complex plant fibers [27]. CAZymes encoded by the rumen microbiome play a pivotal role in facilitating nutrient digestion in ruminants. In this study, differences in rumen microbiome composition were accompanied by functional variations in the microbial community. Regarding gene abundance, yaks exhibited lower levels of carbohydrate‐degrading enzymes than Holstein cows. Metagenomic CAZy profiling revealed that yaks exhibited a greater genomic capacity for lignin and chitin degradation, whereas Holstein cows harbored more genes targeting peptidoglycan, pectin, starch, cellulose, and hemicellulose degradation. The meta‐transcriptomic analysis performed under standardized feeding conditions in this study showed that yaks preferentially expressed starch‐degrading genes at higher levels, whereas Holstein cows actively transcribed enzymes involved in peptidoglycan, pectin degradation. The decoupling of genomic potential (DNA) from functional activity (RNA) suggests that yaks maintain a “standby” reservoir of complex fiber‐degrading enzymes that may be selectively deployed in response to the presence of highly lignified forage. Similar substrate colonization flexibility has been observed in the rumen microbiome of dromedary camels [28] and cattle [29], which rely heavily on lignocellulosic plants and exhibit comparable colonization strategies. In contrast, Holstein cows appeared to sustain a more consistent and broad‐spectrum fiber‐degradation strategy. The ability of the rumen microbiome to colonize various plant‐based substrates is a key functional trait that enables the host to extract energy from diverse and often recalcitrant plant‐derived nutrients. Together, these findings illustrate the presence of host–microbiome co‐adaptation in yaks that primes the rumen ecosystem for the efficient utilization of structurally complex fibers, conferring a distinct advantage in the nutrient‐scarce high‐altitude environments they inhabit [30]. The CAZyme results further showed that *Prevotella* contributed to the decomposition of carbohydrates. *Prevotella* exhibited marked functional variations between yaks and Holstein cows. Specifically, *Prevotella* in yaks appeared to contribute to a narrower but potentially more efficient set of CAZyme functions, whereas the same genus in Holstein cows showed a broader functional spectrum.

KEGG analysis showed that the microbiomes of yaks and Holstein cows significantly differed in various energy metabolism‐related pathways, particularly carbohydrate and energy metabolism. One important finding of this study is that yaks exhibited significantly more active carbon metabolism than Holstein cows at both the metagenomic and meta‐transcriptomic levels. The latter revealed a consistent trend of elevated functional activity in carbon metabolism in yaks, although the difference to Holstein cows was not significant. This suggests that the yak rumen microbiome is functionally inclined toward enhanced carbon utilization, potentially contributing to host adaptation to high‐altitude environments. The central carbon metabolic network, comprising glycolysis, gluconeogenesis, the pentose phosphate pathway, and the TCA cycle, is indispensable for energy metabolism in animals [31, 32]. Based on metagenomic data, the yak rumen microbiome exhibited greater genetic potential for central carbon metabolism compared with that of Holstein cows. Glycolysis provides rapid ATP and pyruvate production under anaerobic conditions. Pyruvate metabolism directs carbon into either downstream fermentation or the TCA cycle, producing additional ATP and reducing equivalents. The pentose phosphate pathway generates NADPH for reductive biosynthesis and antioxidant defense while supplying ribose‐5‐phosphate for nucleotide synthesis [33]. Together, these upregulated routes create a streamlined NADPH‐generating network that maximizes energy extraction and redox balance, enabling yaks to better manage hypoxia and nutrient scarcity at higher altitudes than Holstein cows. In contrast, Holstein cows, adapted to energy‐dense, managed diets under low‐altitude conditions, showed enrichment of complex carbohydrate pathways (starch and sucrose metabolism, pentose and glucuronate interconversions, galactose metabolism, and fructose and mannose metabolism). As Holstein cows are routinely fed a grain‐rich diet, their microbiome has evolved to rapidly ferment readily available polysaccharides, supporting higher energy throughput and production performance. In summary, yaks appear to rely on a focused set of central carbon pathways to enhance the efficiency of carbon use and VFA yield in extreme environments, whereas Holstein cows capitalize on broad complex carbohydrate metabolism to meet their greater energy demands under intensive management. Recent studies have shown that systemic adaptation to chronic hypoxia entails a shift toward catabolic metabolism [34]. The canonical view of hypoxia‐induced metabolism emphasizes the switch from oxidative phosphorylation to increased anaerobic glycolysis in the host as a strategy for coping with oxygen limitation [34]. In line with this paradigm, our results showed a distinct divergence in the rumen energy metabolism pathways between yaks and Holstein cows. Specifically, metagenomic and meta‐transcriptomic analyses revealed that yaks displayed enrichment in glycolysis‐related functions, indicating metabolic adaptation favoring anaerobic energy production. In contrast, Holstein cows exhibited greater enrichment of oxidative phosphorylation pathways, suggesting a higher reliance on oxygen‐dependent ATP production.

VFAs are the primary metabolites produced by rumen microorganisms and serve as the major energy source for the host via gluconeogenesis from propionic acid and lipogenesis from acetic and butyric acids. As reported earlier [6], VFAs were more concentrated in rumen fluid from yaks than that from Holstein cows, with a trend toward a lower A/P ratio (3.5:1 vs. 3.8:1), which was similar to the findings of other studies [35, 36]. As the serum glucose concentrations did not differ between the two species [6], host glucose homeostasis was maintained regardless of variations in fermentation profiles in this study. Similarly, previous studies have reported that yaks maintain stable blood glucose levels even under nutrient‐depleted conditions [37, 38]. The differences in VFAs were supported by the markedly higher digestibility of dietary fiber found in the present yaks compared with the Holstein cows [6]. One aspect that evolved from the present microbiome analyses seems that the methane production pathway, as part of microbial energy metabolism, was enriched in yaks, both in the metagenomic and meta‐transcriptomic results, which corresponds to the higher abundance of archaeal taxa observed in yaks. This observation appears to challenge the prevailing notion of the yak as a low‐methane‐producing (“low‐carbon”) ruminant [39]. Our findings are also consistent with those of previous studies, suggesting that methanogen enrichment in the hindgut of Tibetan populations, compared with that in Han populations, contributes to high‐altitude adaptation [40]. However, this result is not supported by phenotypic data on actual methane emissions, particularly because the abundance of methanogens is not necessarily related to actual methane formation [41]. Collectively, our data support the notion that the enhanced methanogen abundance in yaks may reflect a co‐evolutionary mechanism of microbial and host adaptation to extreme environments, which still requires support from actual methane emission data.

### Characteristics of the rumen epithelium of high‐altitude‐adapted yaks as compared with lowland high‐genetic‐merit Holstein cows

The ruminal epithelium is an absorptive organ and functional mediator between the gut microbiome and host animals, which can regulate host homeostasis. This tissue thus directly affects the health and production performance of ruminants through structural foundations and functions, such as absorption, secretion, metabolism, microbial attachment, and mechanical and immune barriers [42]. A systematic analysis of epithelial tissue cell types and functions is important for revealing the functional mechanisms of the rumen and its response to external stimuli. Based on GO functional enrichment analysis, we showed that the yak epithelium, compared to the Holstein cow epithelium, was depleted in energy metabolism‐related pathways, such as the carbohydrate derivative biosynthetic, organic acid metabolic, and carboxylic acid metabolic processes, along with microbial functional features that support the rapid fermentation of energy‐dense feeds. Therefore, yaks and Holstein cows exhibited distinct energy utilization strategies at the level of rumen epithelial metabolism, similar to those found in the rumen microbiome, reflecting divergent host–microbiome co‐adaptations to their respective ecological niches. Consistently, yaks showed significantly lower expression levels of VFA transporters, including *SLC16A1* (also known as *MCT1*) and *SLC12A7*, which are involved in the uptake of lactate and VFAs, than Holstein cows. For Holstein cows, this expression pattern supports rapid fermentation turnover, efficient energy absorption, and acid–base regulation, which is consistent with the demands of intensive, nutrient‐rich feeding regimes. Instead, yaks demonstrated enhanced expression levels of genes involved in ketone body metabolism (e.g., *ACAT1*, *HMGCS2*, *HMGCL*, and *BDH1*) and mitochondrial fatty acid β‐oxidation, and elevated levels of transporters such as *SLC16A9*, *SLC4A7*, and *SLC22A23*, which mediate the transport of ketone bodies and organic acids. These patterns indicate a metabolic shift in yaks toward secondary metabolism and recycling of energy‐dense fermentation products, such as acetate and butyrate, through ketone body synthesis and oxidative pathways. Pathway scoring further confirmed that ketone body synthesis and fatty acid beta‐oxidation were significantly upregulated in yaks, whereas VFA catabolism was stronger in Holstein cows.

This differential gene expression indicates that yak optimizes metabolic energy generation via fatty acid beta‐oxidation metabolism and ketone cycling, a strategy well‐suited to sustaining energy supply and maintaining redox balance under chronic hypoxia and nutrient limitation at high altitudes. In contrast, functional partitioning in Holstein cows relies on rapid VFA uptake to meet high energy demands. In most mammals, including humans, the liver is the major site of ketone body synthesis (acetoacetate and β‐hydroxybutyrate). However, the enrichment of *ACAT1*, *HMGCS2*, *HMGCL*, and *BDH1* suggests enhanced *de novo* ketone body synthesis in the yak rumen epithelium [43], where *HMGCS2* is assumed to act as the rate‐limiting enzyme in ruminal ketone body synthesis and *BDH1* catalyzes the reversible conversion between acetoacetate and β‐hydroxybutyrate, the two principal ketone bodies [44]. At the level of rumen epithelial cell metabolism, yaks exhibited enhanced ketone body synthesis and fatty acid β‐oxidation, as evidenced by the upregulation of genes such as *BDH1* (3‐hydroxybutyrate dehydrogenase) and *ACAT1*. In contrast, the Holstein cows’ rumen epithelial cells exhibited significantly higher *SLC16A1* expression [45], indicating a stronger capacity for VFA transport across the intestinal epithelium. These differences indicate distinctly different energy utilization strategies between the two species, reflecting the co‐evolution of the host and microbiome to optimize energy utilization in their respective ecological niches.

### Functional validation of central carbon metabolism differences via rumen fluid transplantation

In terms of microbial composition and function, the rumen microbiome of yaks is more adapted to coarse forage and efficient energy supply, thereby conferring better adaptation to high‐altitude environments. To verify the environmental adaptability of the yak's rumen, its microbiome was transplanted into dairy cows to investigate its effects on production performance and rumen microbial communities. Indeed, manipulation of the gut microbiome has emerged as a promising strategy for modulating host metabolism [46, 47], restoring microbial ecosystem functions [48], and enhancing the production traits of livestock [16, 49]. The goal of this study was to counteract hypoxia‐induced energy deficiency by transplanting rumen fluid from a high‐altitude‐adapted species (yak) to one not bred for this purpose (Holstein cows).

Rumen fluid transplantation from yaks to Holstein cows resulted in a significant increase in the milk, protein, and lactose yields on Day 7 post‐transplantation. This highlights the capacity of the yak‐derived microbiome to enhance energy‐demanding milk synthesis, particularly milk yield, likely due to improved ruminal fermentation efficiency and total tract digestibility in yaks compared with Holstein cows, as previously demonstrated by Zhang et al. [6]. As energy deficiency is a key element in adapting to hypoxia at high altitudes, these findings provide strong evidence that the adapted rumen microbiome indirectly contributes to high‐altitude tolerance by supporting energy metabolism pathways.

In adult ruminants, stable modification of the rumen microbiome via exogenous interventions remains a major challenge because of host‐specific selective pressures, including diet composition, immune regulation, and epithelial signaling, which drive the restoration of the original microbial community [50]. Accordingly, the increase in the expression of pathways noted 6 h post‐transplantation declined on Day 7 and Day 28, marking a progressive transition in microbial metabolic capacity. In contrast, pathways involved in the degradation of upstream carbon substrates, such as starch and sucrose metabolism, amino sugar and nucleotide sugar metabolism, galactose metabolism, fructose and mannose metabolism, and pentose and glucuronate interconversion, gradually increased with time post‐transplantation. This pattern suggests a functional reversion of the microbial community toward the native Holstein cows' state, shifting from an initial high‐efficiency energy conversion profile to a broader substrate‐degrading metabolic strategy.

The transplantation experiment was also useful for functionally validating the microbial metabolic differences observed between the yaks and Holstein cows. Metagenomic profiling demonstrated that, within the first 6 h post‐transplantation, the yak‐derived microbiome caused elevated expression of pathways related to glycolysis/gluconeogenesis; pyruvate, butanoate, and propanoate metabolism; and the TCA cycle, suggesting an early phase enhancement of energy metabolism. Seven days post‐transplantation, metagenomic analyses revealed pronounced enrichment of microbial pathways related to central carbon metabolism, including glycolysis (M00002), the pentose phosphate pathway (M00004 and M00007), and the Entner–Doudoroff pathway (M00008). These changes in the core metabolic modules underpin ATP production, reducing power (NADPH) generation, and biosynthetic precursor synthesis, indicating an increase in microbial metabolic activity and carbon flux integration. This transient upregulation mirrors the distinct metabolic traits of the yak rumen microbiome and is likely an adaptive feature of high‐altitude microbial communities that have evolved under hypoxic, cold, and nutrient‐limited conditions.

The period between Day 7 and Day 14 post transplantation possibly represents the “metabolic efficiency window” [51]. Within this window, the elevation of core energy pathways, such as glycolysis, pyruvate metabolism, and the TCA cycle, persisted, supporting enhanced microbial carbon utilization and energy release. Collectively, these results provide direct functional evidence that the yak‐derived rumen microbiome has adaptive metabolic configurations tailored to high‐altitude environments that can be transiently transferred to lowland hosts. Although no long‐term structural colonization was achieved, the temporary enhancement of central carbon metabolism represents a valuable mechanism for boosting host energy metabolism and productivity. This “transient metabolic advantage” could be the basis for novel microecological strategies to improve dairy cow performance, particularly in high‐altitude environments or during periods of energy deficiency caused by other factors. This finding suggests that, although yak‐derived microbial species may not achieve long‐term structural colonization in the Holstein cow rumen, they can transiently enhance central energy metabolism, providing more efficient energy conversion support to the host and improving its productive performance. Future research should focus on practical and sustainable approaches, such as targeted dietary interventions, probiotics, or microbial consortia supplementation, that could mimic the beneficial effects observed in the present study with rumen fluid transplantation and such studies should also be carried out over extended periods of time.

## CONCLUSIONS

To the best of our knowledge, this study provides the first comprehensive energy‐centered comparison of rumen ecology in long‐term high‐altitude‐adapted yaks compared with lowland Holstein cows with high genetic merit. We demonstrated that yak and Holstein rumen microbiomes differ in their genomic and transcriptomic repertoires for central carbon metabolism and energy pathways, reflecting long‐term selection pressures imposed by hypoxia and, possibly, other high‐altitude conditions. The yak rumen epithelial cells exhibited upregulated expression of genes involved in ketone body biosynthesis and fatty acid beta‐oxidation, supporting more efficient energy utilization under oxygen‐limited conditions. We further validated the functional importance of these microbial traits, as the transfer of the yak rumen microbiome by rumen fluid transplantation enhanced milk production in Holstein cows without perturbing systemic metabolic markers. Accordingly, gastrointestinal microbial species may assist in counteracting energy deficiency in hypoxic environments. Even though the metabolic advantage was partially transient within 14 days post‐transplantation, our study offers a novel framework for future microecological strategies related to the role of gastrointestinal microbiome regulation in high‐altitude adaptation. Collectively, these findings delineate a coordinated host–microbiome specialization in central carbon metabolism and ketone body synthesis. This study also lays a theoretical foundation for the future exploration of gut microbiome‐mediated energy homeostasis in other animal models and suggests intriguing possibilities for translational research on human adaptation to hypoxia. A limitation of our experimental designs is that it was not possible to disentangle the influence of the microbiome from a possible additional influence of host genetics, going beyond the microbiome's influence.

## METHODS

### Experimental designs and sampling

This study was conducted on yaks and Holstein cows born and housed at the Lhasa Jingtu Company in Tibet. The study design is presented in Figure 6. Feed was always offered as a total mixed ration with all diet ingredients being included in constant proportions throughout each experiment, a procedure which guaranteed minimal selection by the animals and thus a consistent diet and microbial composition. The diet was provided *ad libitum* and renewed twice daily. Rumen fluid samples were collected orally using a stomach tube, with the first 20 mL discarded, and the remainder was filtered through four layers of cheesecloth. Subsequently, the rumen fluid was centrifuged at 12,000 × *g* for 10 min at 4°C. For rumen microbiome analysis, samples were immediately frozen in liquid nitrogen (−196°C).

**Figure 6:**
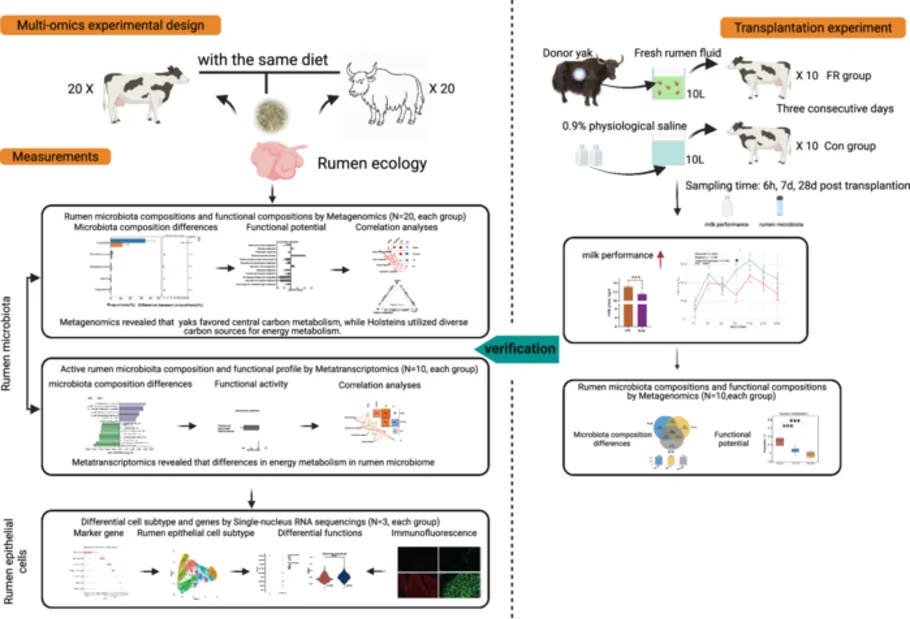
Schematic representation of the study cohort design and analysis process flow.

#### Comparison of the microbiome of yaks with that of a high genetic merit lowland cattle breed (Experiment 1)

Twenty mid‐lactating yaks (stage of lactation, 50 ± 12 days) and twenty mid‐lactating Holstein cows (stage of lactation, 108 ± 17 days; means ± standard deviation) were enrolled in a completely randomized design. The experiment lasted 44 days (April–May 2023), with sampling on Day 44. All animals received an identical diet (forage:concentrate = 2:1). It was composed (g/kg dry matter) of oat hay, 263.0; alfalfa hay, 127.7; whole corn silage, 251.7; concentrate, 325.4; soybean meal, 17.6; sodium bicarbonate, 7.3; and palm oil fatty acid powder, 7.3. The analyzed composition is provided in detail in Zhang et al. [6]. Despite the physiological differences in nutrient utilization and metabolism between yaks and Holstein cows, feeding exactly the same diet was considered most important, as it prevented a dietary influence on the gut microbiome and thus facilitated the comparison as best as possible. Rumen fluid from all animals was collected before the morning feeding and was subjected to metagenomic analysis, and 10 samples from yaks and Holstein cows were subjected to meta‐transcriptomic analysis. Finally, three yaks and three Holstein cows were randomly selected to collect ventral rumen epithelial tissue by surgery. Prior to surgery, the dorsoventral side of the skin above the left rumen side was shaved and disinfected with povidone–iodine and medical alcohol. A small incision was made with a scalpel to obtain approximately 200 mg of rumen tissue (approximately the size of a soybean), which was washed twice with PBS, immediately placed into a tissue preservation solution, and sent to a sequencing company for sequencing within 12 h. The opening of both the rumen and skin was sewn and disinfected immediately afterwards. Using three animals or fewer is common in snRNA‐seq [45] with *n* = 3 allowing for analysis of variance to compare yaks and Holstein cows. More details can be found in Zhang et al. [6].

#### Effect of transplanting rumen fluid from yaks to a high‐bred lowland cattle breed (Experiment 2)

A 28‐day experiment was conducted from May to June 2023 on the Holstein cow farm. Rumen fluid transplantation was performed as described by DePeters and George [52]. Ten mid‐lactating primiparous yaks with similar body weights were selected from farm yaks. These yaks were fitted with rumen cannulas 6 weeks before the start of the experiment to serve as donor animals and were moved to temporary pens on the Holstein cow farm 15 days before the start of the experiment. Until the start of and during transplantation experiment, yak donors were fed the diet that all yaks received at that farm. On a dry matter basis, the diet was composed of wheat straw, 42%; whole‐plant corn silage, 6%; concentrate, 51%; and sodium bicarbonate, 1%. Rumen fluid for transplantation was obtained by manually removing, squeezing, and filtering the rumen contents from 10 yaks. The resulting clear rumen fluid from the 10 yaks was mixed and placed in a sealed insulated container (39°C), and the remainder was returned to the yak rumens. Ten Holstein cows (FR group) were inoculated via an oral tube with 10 L/cow of fresh mixed yak rumen fluid. By random division of the 20 experimental Holstein cows, another group of 10 Holstein cows was formed, which was inoculated with 10 L/cow of 0.9% physiological saline (group Con). This procedure was repeated before morning feeding for three consecutive days. The entire daily transplantation process was completed in 30 min. Rumen fluid was sampled at 6 h, and on Day 7 and Day 28 after completing the 3‐day rumen fluid transplantation procedure. These time points were chosen to capture distinct stages of microbial colonization. In this respect, 6 h after transplantation was considered to represent the immediate response phase, allowing the rumen microbiome of present and transplanted rumen fluid to settle, 7 days after transplantation to reflect early colonization dynamics, and 28 days after transplantation to afford early indications of a likely equilibrium establishing and to illustrate whether or not the transplanted rumen microbiome persisted. Milk recording and sampling started first on Day 1 post‐transplantation.

### Metagenomic sequencing in rumen fluid

Majorbio Bio‐Pharm Technology Co., Ltd. performed the microbial sequencing. Total genomic DNA was extracted from 2 mL of rumen fluid using the MagAtract PowerSoil Pro DNA Kit (Qiagen), according to the manufacturer's instructions. The concentration and purity of the extracted DNA were determined using a TBS‐380 and NanoDrop 2000, respectively. DNA quality was assessed using 1% agarose gel.

The DNA extract was fragmented to an average size of approximately 400 bp using a Covaris M220 (Gene Company Limited) for paired‐end library construction. This library was constructed using a NEXTFLEX Rapid DNA‐Seq Kit (BioScientific) on an Illumina NovaSeq. 6000 system (Illumina Inc.).

The raw sequencing reads were trimmed of adaptors, and low‐quality reads (length < 50 bp, quality value < 20, or having N bases) were removed using fastp [53]. Reads were aligned to the cattle reference genome using ARS‐USD 1.2, and the hits associated with the reads and their mated reads were removed. Quality‐filtered data were assembled using MEGAHIT [54]. Contigs with a length of ≥300 bp were selected as the final assembly result. Open‐reading frames (ORFs) from each assembled contig were predicted using Prodigal (https://github.com/hyattpd/Prodigal, version 2.6.3) and those with a length of ≥100 bp ORFs were retrieved. A non‐redundant gene catalog was constructed using the method described by Fu et al. [55] with 90% sequence identity and 90% coverage. Gene abundance in a certain sample was estimated using SOAPaligner [56] with 95% identity. We used DIAMOND v2.0.13 with the blastp algorithm and set the threshold at e‐value ≤ 1e^−05^, aligned against the NR database (version 202209 for Experiment 1 and version 20241007 for Experiment 2).

Abundance was calculated using TPM. The functionality of the non‐redundant genes was obtained by annotation using mmseqs. 2 in the “search” mode against the KEGG and CAZy protein databases. The distribution of CAZymes and KEGG pathways was visualized using the ggplot2 package in R.

### Meta‐transcriptomic sequencing in rumen fluid

Total RNA was isolated and purified using a soil RNA extraction kit (Majorbio) following the manufacturer's instructions. The integrity and quantity of the extracted RNA were measured using a NanoDrop 2000 spectrophotometer (Thermo Scientific) and an Agilent 5300 Bioanalyzer (Agilent Technologies).

Fastp [48] was used to trim the 3' and 5' end sequences and remove the low‐quality reads. The reads were aligned with the SILVA SSU and SILVA LSU databases using the SortMeRNA software (http://bioinfo.lifl.fr/RNA/sortmerna/, version 2.1b) to remove rRNA fragments. The optimized sequences were assembled using Trinity [57]. Transcripts with lengths of ≥300 bp were selected from the assembled results. CD‐HIT [50] was used to cluster and remove redundant transcriptomes with a length of ≥300 bp that were obtained from all samples. The longest gene in each class was used as a representative sequence to construct a UniGene set for subsequent species and functional annotation analyses. Clean reads were compared with the UniGene set to calculate gene expression. Diamond [58] was used to compare the amino acid sequences of the UniGene set with the NR database (version 20230609) (BLASTP, with the comparison parameter setting expected value e‐value as 1e^−5^), and species annotation was obtained through the taxonomic information database corresponding to the NR database. The species expression abundance was calculated using the sum of the gene expression abundances corresponding to each species. The abundances of KEGG and CAZy protein were normalized to TPM for downstream analyses.

### Single‐nuclei RNA sequencing in rumen epithelium

Flash‐frozen was homogenized using a Dounce homogenizer in lysis buffer with 1 mM DTT and 1 U/µL RNase inhibitor, and incubated on ice for 5 min. The suspension was filtered through a 40‐μm filter to remove debris and centrifuged at 4°C at 500 × *g* for 5 min. After the nuclei were resuspended in 300 μL lysis buffer and 300 μL RB buffer in a 2‐mL tube, the mixture was centrifuged by density gradient to separate the nuclei from the cell debris, and the intermediate layer containing nuclei was collected and washed with RB buffer. Finally, the cell numbers and viability were assessed using an automatic cell counter.

Cell suspensions (300–600 living cells per microliter determined by Count Star) were loaded onto a Chromium Single‐Cell Controller (10× Genomics) to generate single‐cell gel beads in emulsion (GEMs) using the Single Cell 3' Library and Gel Bead Kit V2 (10× Genomics, 120237) and Chromium Single Cell A Chip Kit (10× Genomics, 120236) according to the manufacturer's protocol. Briefly, single cells were suspended in 0.04% BSA–PBS. Approximately 22,000 cells were added to each channel, with a target cell recovery of 20,000 cells. Captured cells were lysed, and the released RNA was barcoded through reverse transcription in individual GEMs. GEMs were reverse‐transcribed in a C1000 Touch Thermal Cycler (Bio Rad) programmed at 53°C for 45 min, 85°C for 5 min, and maintained at 4°C. After reverse transcription, single‐cell droplets were broken, and single‐stranded cDNA was isolated and cleaned using a Cleanup Mix containing DynaBeads (Thermo Fisher Scientific). The cDNA was generated and amplified, and its quality was assessed using an Agilent 4200.

SnRNA‐seq libraries were prepared using the Single‐Cell 3Library Gel Bead Kit V2 following the manufacturer's instructions. Sequencing was performed on an Illumina NovaSeq. 6000 with a sequencing depth of at least 100,000 reads per cell and paired ends of 150 bp (PE150).

Raw FASTQ files were mapped to the reference genome (Bos_taurus.ARS‐UCD1.2) using CellRanger‐7.0.0 (10× Genomics). To create Cell Ranger‐compatible reference genomes, the references were rebuilt according to the instructions provided by 10× Genomics to perform alignment, filtering, barcode counting, and UMI counting. Following alignment, digital gene expression (DGE) matrices were generated for each of the samples. Merged 10× Genomics DGE files were generated using the aggregation function of the Cell Ranger pipeline. All cells obtained in different batches were merged, and the data were normalized by equalizing read depth among the libraries. The result was a matrix of all the cells, barcodes, and features.

### Immunofluorescence measurements on paraffin‐embedded sections

Rumen tissue slides (5 μm) were deparaffinized in xylene (China National Pharmaceutical Group Corp.) for 15 min twice, rehydrated using a graded ethanol series (100%, 85%, and 75%; 5 min each), and rinsed in distilled water. Antigen retrieval was performed by immersing the slides in EDTA buffer (pH 9.0) and heating in a microwave oven (medium power, 8 min; rest, 8 min; medium–low power, 10 min). The slides were cooled to room temperature and washed thrice with PBS (pH 7.4) on a rocker (5 min each). The sections were encircled with a hydrophobic barrier pen and treated with an autofluorescence quenching reagent for 5 min, followed by a 10‐min rinse under running tap water. To block non‐specific binding, the sections were incubated with 5% normal goat serum in PBS for 1 h at room temperature. Primary antibodies—*ACAT1* (rabbit polyclonal, Sanying Biotechnology, Wuhan, China; 1:200), *HMGCS2* (rabbit polyclonal, Abcam, Cambridge, UK; 1:200), *HMGCL* (rabbit polyclonal, Sanying Biotechnology, Wuhan, China; 1:200), *BDH*1 (rabbit polyclonal, Sanying Biotechnology, Wuhan, China; 1:200)—were diluted in PBS and applied overnight at 4°C in a humidified chamber. After three washes in PBS (5 min each), the sections were incubated with CY3‐conjugated goat anti‐rabbit IgG for 50 min in the dark at room temperature. After three washes with PBS, the nuclei were counterstained with DAPI. The sections were washed thrice with PBS and mounted on an antifade medium. Fluorescence images were acquired using an epifluorescence microscope equipped with CY3 and DAPI filter sets, an objective magnification lens, and a numerical aperture. Rumen tissue sections were visualized and imaged using an Olympus CX23 light microscope.

### Bioinformatic and statistical analysis

#### Statistical analysis of the microbiome data

The differences between yaks and Holstein cows in the alpha diversity indices (Shannon, Chao1, and Simpson) were tested using a Wilcoxon rank‐sum test and visualized using GraphPad software (version 9). PCoA of Bray–Curtis distance and linear discriminant analysis Effect Size were performed on the free online Majorbio Cloud Platform (www.majorbio.com). Beta diversity differences between groups were assessed using permutational multivariate analysis of variance (PERMANOVA) with 999 permutations, implemented via the “adonis” function in the vegan package for R. The differences between microbial groups were tested using linear discriminant analysis Effect Size analysis for pairwise comparisons. This analysis shows the differential bacterial taxa with LDA > 2 and *p* < 0.05. The R package “Psych” was used to perform the Spearman's rank correlation analysis between production traits and rumen microbial species, and visualization was conducted using the ggplot2 package. The relative abundance of microbial taxa and TPM of metagenomic and meta‐transcriptomic functions were compared between the two groups using the Wilcoxon rank‐sum test. The mediating effect model was used to construct a regulation model (rumen microbial taxa–metabolites–host energy metabolism) using process plug‐ins in SPSS. The *p‐*values were adjusted using the false discovery rate (using the BH method of the stats package in R) [59].

#### Uniform manifold approximation and projection—visualization and determination of the major cell types

Gene expression analysis and cell type identification were performed using the Seurat V5.1.0 pipeline (http://satijalab.org/seurat/) after filtering and normalization using another R toolkit for single‐cell transcriptomics [60]. As the data were normalized, they were loaded into Seurat without normalization, scaling, or centering. Along with expression data, metadata for each cell were collected, including information such as nCount_RNA, nFeature_RNA, and cell type information. Next, highly variable genes were identified and used as input for dimensionality reduction using principal component analysis. The resulting PCs and correlated genes were examined to determine the number of components to be included in the downstream analysis. UMAP was performed on the first 50 principal components to visualize cells in a two‐dimensional space. The Seurat function FindAllMarkers was used to identify DEGs in each cluster. Finally, the cell clusters were annotated to known biological cell types using canonical markers. “VFA catabolic process” and “ketone body biosynthetic process” gene sets were obtained from the MSigDB database. Short‐chain (Volatile) fatty acid genes (“*ACADS*,” “*MCEE*,” “*MMAA*,” “*MMUT*,” “*PCCA*,” “*PCCB*,” “*PCK1*,” “*PCK2*,” “*PHYH*”); ketone body biosynthesis genes <‐ c (“*AACS*,” “*ACAT1*,” “*ACSS3*,” “*BDH1*,” “*BDH2*,” “*HMGCL*,” “*HMGCLL1*,” “*HMGCS2*”) according to Xue. et al. (2022) [45]. Comparison of fatty acid beta‐oxidation with the GO gene set. The signature score of each gene set for each cell type was computed using the “AddModuleScore” function in the Seurat R package. Differences in signature scores across cell types were evaluated using a two‐sided Wilcoxon rank‐sum test.

#### Analysis of milk yield of transplanted and control Holstein cows

Data on milk, milk protein, and milk lactose yields were statistically analyzed using a linear mixed model (LMM), reading *Y*_*i*×*j*×*k*_ = *μ* + *D_i_* + *T_j_* + *D_i_* × *T_j_* + *C_k_* + *e*_*i*×*j*×*k*_, where *Y*_*i*×*j*×*k*_ = dependent variable; *μ* = population mean; *D_i_* = sampling time fixed effect; *T_j_* = treatment fixed effect; *D_i_* × *T_j_* = treatment × time interaction effect; *C_k_* = cow random effect; *e*_*i*×*j*×*k*_ = residual error. LMM analysis was performed in the R package “lme4” (v1.1.31).

## AUTHOR CONTRIBUTIONS

**Xinyu Zhang**: Methodology; software; conceptualization; investigation; data curation; formal analysis; writing—original draft; visualization. **Senlin Zhu**: Visualization; validation; formal analysis; software; methodology; writing—original draft. **Michael Kreuzer**: Validation; visualization; writing—original draft; writing—review and editing; methodology; software. **Shoukun Ji**: Writing—original draft; methodology. **Wei Wang**: Writing—review and editing; conceptualization. **Yanliang Bi**: Writing—original draft. **Shengli Li**: Writing—review and editing; supervision; project administration; funding acquisition; conceptualization; resources. All authors have read the final manuscript and approved it for publication.

## CONFLICT OF INTEREST STATEMENT

The authors declare no conflicts of interest.

## ETHICS STATEMENT

The animal care methods and procedures used in both experiments were approved by the Institutional Animal Care and Use Committee (protocol number: AW12203202‐1‐1) of the College of Animal Science and Technology, China Agricultural University, Beijing, China.

## Supporting information


**Figure S1.** Differences between Holstein cows (H) and yaks (Y) in alpha diversity based on Wilcoxon rank sum test in rumen microbiome by metagenomic analysis (n = 20 per group; Experiment 1). All microbial species (A), archaea (B), and bacteria (C).
**Figure S2.** Differences between Holstein cows (H) and yaks (Y) in rumen microbial composition and its association with end products of ruminal fermentation, based on metagenomic analysis (*n* = 20 per group; Experiment 1). (A) Pie chart of unique microbe species and groups of taxa of archaea and bacteria for Holstein cows and yaks. (B) Differences in common archaeal species composition between Holstein cows and yaks determined by Linear discriminant analysis Effect Size. Heatmap of correlations between rumen‐specific archaea (C for Holstein cows, D for yak group; |*r*| > 0.6) as well as bacteria (E for Holstein cows, F for yak group; |*r*| > 0.7) and end products of ruminal fermentation in Holstein cows and yaks, Heatmap of correlation between the abundance of individual archaeal species common to Holstein cows and yaks and end products of ruminal fermentation in Holstein cows (G) and yaks (H). Heatmap of correlations between the abundance and individual bacterial species common to Holstein cows (I) and yaks (J) and end products of ruminal fermentation. A/P: acetate‐to‐propionate ratio.
**Figure S3.** Differences in CAZy and KEGG functional profiles between Holstein cows (H) and yaks (Y), based on metagenomic analysis (*n* = 20 per group; Experiment 1). Pie charts of CAZyme composition at the class and family levels for Holstein cows (A) and yaks (B). Functional differences of CAZyme at the class level (C) and family level (D) between Holstein cows and yaks with Wilcoxon rank sum test. (E) Carbohydrate degradation enzyme function differences with Wilcoxon rank sum test. (F‐G) Fold changes of Kyoto Encyclopedia of Genes and Genomes (KEGG) pathways identified in the metagenomes. Pathways were sorted for amino acid metabolism (F), and lipid metabolism (G); The Wilcoxon rank‐sum test was used for mean comparison. *adjusted *p* < 0.05; **adjusted *p* < 0.01; ***adjusted *p* < 0.001.
**Figure S4.** Chart showing the established mediating effect models.
**Figure S5.** Differences between the Holstein cows (H) and the yaks (Y) in rumen microbial composition and function in the rumen fluid as determined by metatranscriptome analysis (*n* = 10 per group; Experiment 1). Differences between Holstein cows and yaks in alpha diversity of all microbial species (A) archaea (B) and bacteria (C) in rumen microbiome. (D) Pie chart of unique rumen microbe species and groups of taxa of archaea and bacteria for Holstein cows and yaks. Differences in common archaeal species composition (E) between Holstein cows and yaks determined by Linear discriminant analysis Effect Size. (F) Carbohydrate degradation enzyme function differences with Wilcoxon rank sum test. (G, H) Fold changes of KEGG pathways identified in the metagenomes. Pathways were sorted for amino acid metabolism (G), and lipid metabolism (H); The Wilcoxon rank‐sum test was used for mean comparison. *adjusted *p* < 0.05; **adjusted *p* < 0.01; ***adjusted *p* < 0.001.
**Figure S6.** Differences between the Holstein cows (H) and the yaks (Y) as illustrated by a rumen epithelial single cell map (*n* = 3 per group; Experiment 1). (A) Distribution of cells in each sample. (B) Distribution of cells in each cell clustering. Marker gene expression in single cell type of Holstein cows (C)and yaks (D). Uniform manifold approximation and projection maps of cell types from Holstein cows (E) and yaks (F) rumen samples, with each cell color indicating the associated cell subtypes. Sn = single‐nucleus RNA sequencing.
**Figure S7.** Cell subtype correlations and functional differences between Holstein cows (H) and the yaks (Y) (*n* = 3 per species; Experiment 1). (A) Heat map of the similarities between Holstein cows and yaks in cell subtypes, where the redder the color the stronger the correlation. (B) Differences between Holstein cows and yaks in the top 15 gene ontology function pathways. (C‐E) scores of different cell subtypes from groups Holstein cows and yaks in the VFA catabolic process, ketone body biosynthesis metabolism, and fatty acid beta‐oxidation. Sn = single‐nucleus RNA sequencing. E = epithelial cell.
**Figure S8.** Effect of yak rumen fluid transplantation on microbiome composition and functional profiles in Holstein cows (FR= Fresh rumen fluid group) at 6 h, and on Day 7 and Day 28 post transplantation. (*n* = 10 per group; Experiment 2) (A) Key differential genera at 6 h, and on Day 7 and Day 28 after rumen fluid transplantation. (B) Overview of module differences (*p* < 0.05) between Holstein cows (H) and yaks (Y) in metagenomic and metatranscriptomic profiles, alongside alterations in carbon metabolism modules following rumen fluid transplantation as determined the using Kruskal–Wallis test. (C) Differences in carbon metabolism modules following rumen fluid transplantation based on Wilcoxon rank sum test.

**Table S1.** Differences in rumen fluid properties between mid‐lactating yaks and Holstein cows kept at high altitude (Experiment 1).
**Table S2.** Sequencing data from metagenomic sequencing (Y = yak, H = Holstein cow) (Experiment 1).
**Table S3.** Taxonomic composition at the domain level based on metagenomic analysis (Experiment 1).
**Table S4.** Differentially expressed KEGG level 3 pathways identified by Wilcoxon rank‐sum test based on metagenomics (Experiment 1).
**Table S5.** Differentially expressed KEGG level 1 pathways identified by Wilcoxon rank‐sum test based on metagenomics (Experiment 1).
**Table S6.** Sequencing data from metatranscriptomics (Y = yak, H = Holstein cow) (Experiment 1).
**Table S7.** Differentially expressed genera (transcripts per million mapped reads (TPM)) identified by Wilcoxon rank‐sum test based on metatranscriptomic profiling (Experiment 1).
**Table S8.** Differentially expressed KEGG level 3 pathways identified by Wilcoxon rank‐sum test based on metatranscriptomic profiling (Experiment 1).
**Table S9.** Sequencing data from metagenomic sequencing in transplantation experiment (FR = Fresh rumen fluid group, Con = Control group) (Experiment 2).

## Data Availability

The raw data associated with this project are deposited in the NCBI database (Accession Numbers: PRJNA1299708 (https://www.ncbi.nlm.nih.gov/bioproject/PRJNA1299708), PRJNA1291843 (https://www.ncbi.nlm.nih.gov/bioproject/?term=PRJNA1291843), PRJNA1279085 (https://www.ncbi.nlm.nih.gov/bioproject/?term=PRJNA1279085), PRJNA1280711 (https://www.ncbi.nlm.nih.gov/bioproject/?term=PRJNA1279085)). The data and scripts used are saved in GitHub (https://github.com/XinyuZhang001/iMeta_1040). Supplementary materials (figures, tables, graphical abstract, slides, videos, Chinese translated version, and update materials) may be found in the online DOI or iMeta Science http://www.imeta.science/. The data that support the findings of this study are available in the supplementary material of this article.

## References

[1] Peng, Xuefeng , St. Elmo Wilken , Thomas S. Lankiewicz , Sean P. Gilmore , Jennifer L. Brown , and John K. Henske . 2021. “Genomic and Functional Analyses of Fungal and Bacterial Consortia That Enable Lignocellulose Breakdown in Goat Gut Microbiomes.” Nature Microbiology 6(4): 499–511. 10.1038/s41564-020-00861-0 PMC8007473 33526884

[2] Groussin, Mathieu , Florent Mazel , Jon G. Sanders , Chris S. Smillie , Sébastien Lavergne , Wilfried Thuiller , and Eric J. Alm . 2017. “Unraveling the Processes Shaping Mammalian Gut Microbiomes Over Evolutionary Time.” Nature Communications 8(1): 14319. 10.1038/ncomms14319 PMC5331214 28230052

[3] Flint Harry, J . 2004. “Polysaccharide Breakdown by Anaerobic Microorganisms Inhabiting the Mammalian Gut, in Advances in Applied Microbiology 56, pp. 89–120. Elsevier 10.1016/S0065-2164(04)56003-3 15566977

[4] Qiao, G. H. , T. Shao , C. Q. Yu , X. L. Wang , X. Yang , X. Q. Zhu , and Y. Lu . 2013. “A Comparative Study at Two Different Altitudes With Two Dietary Nutrition Levels on Rumen Fermentation and Energy Metabolism in Chinese Holstein Cows.” Journal of Animal Physiology and Animal Nutrition 97(5): 933–941. 10.1111/j.1439-0396.2012.01339.x 22882230

[5] Kulyar, Md. F. , Quan Mo , Shah Nawaz , and Jiakui Li . 2025. “High Altitude Microbiome: Insight into Yak Gut Microbiota and Its Nutritional and Functional Involvement for Food Systems.” Trends in Food Science & Technology 159: 104897. 10.1016/j.tifs.2025.104897

[6] Xinyu, Zhang , Michael Kreuzer , Mutian Niu , and Shengli Li . 2025. “Differences in Milk Production Efficiency and Physiology Between Lactating Holstein Cows and Yaks Born and Kept at About 4000 m of Altitude When Fed the Same Diet.” Journal of Animal Physiology and Animal Nutrition jpn 109: 14122. 10.1111/jpn.14122 PMC12451412 40261988

[7] Qiu, Qiang , Guojie Zhang , Tao Ma , Wubin Qian , Junyi Wang , Zhiqiang Ye , Changchang Cao , et al. 2012. “The Yak Genome and Adaptation to Life at High Altitude.” Nature Genetics 44(8): 946–949. 10.1038/ng.2343 22751099

[8] Liu, Xinfeng , Wenyu Liu , Johannes A. Lenstra , Zeyu Zheng , Xiaoyun Wu , Jiao Yang , Bowen Li , et al. 2023. “Evolutionary Origin of Genomic Structural Variations in Domestic Yaks.” Nature Communications 14(1): 5617. 10.1038/s41467-023-41220-x PMC10509194 37726270

[9] Xin, Jinwei , Zhixin Chai , Chengfu Zhang , Qiang Zhang , Yong Zhu , Hanwen Cao , Cidan Yangji , et al. 2020. “Methylome and Transcriptome Profiles in Three Yak Tissues Revealed That DNA Methylation and the Transcription Factor ZGPAT Co‐Regulate Milk Production.” BMC Genomics 21(1): 731. 10.1186/s12864-020-07151-3 PMC7576800 33081725

[10] Zhang, Zhigang , Dongming Xu , Li Wang , Junjun Hao , Jinfeng Wang , Xin Zhou , Weiwei Wang , et al. 2016. “Convergent Evolution of Rumen Microbiomes in High‐Altitude Mammals.” Current Biology 26(14): 1873–1879. 10.1016/j.cub.2016.05.012 27321997

[11] Li, Bin , Yuangang Yang , Bo Xu , Pengfei Song , Feng Jiang , Hongmei Gao , Zhenyuan Cai , Haifeng Gu , and Tongzuo Zhang . 2025. “Comparative Macrogenomics Reveal Plateau Adaptation of Gut Microbiome in Cervids.” BMC Biology 23(1): 154. 10.1186/s12915-025-02269-w PMC12139185 40468269

[12] Zhao, Fangfang , Lili Yang , Tao Zhang , Daohua Zhuang , Qunfu Wu , Jiangkun Yu , Chen Tian , and Zhigang Zhang . 2023. “Gut Microbiome Signatures of Extreme Environment Adaption in Tibetan Pig.” npj Biofilms and Microbiomes 9(1): 27. 10.1038/s41522-023-00395-3 PMC10209067 37225687

[13] Su, Qian , Dao‐Hua Zhuang , Yu‐Chun Li , Yu Chen , Xia‐Yan Wang , Ming‐Xia Ge , Ting‐Yue Xue , et al. 2024. “Gut Microbiota Contributes to High‐Altitude Hypoxia Acclimatization of Human Populations.” Genome Biology 25(1): 232. 10.1186/s13059-024-03373-w PMC11350960 39198826

[14] Wu, Jia‐Jin , Senlin Zhu , Fengfei Gu , Teresa G. Valencak , Jian‐Xin Liu , and Hui‐Zeng Sun . 2022. “Cross‐Tissue Single‐Cell Transcriptomic Landscape Reveals the Key Cell Subtypes and Their Potential Roles in the Nutrient Absorption and Metabolism in Dairy Cattle.” Journal of Advanced Research 37: 1–18. 10.1016/j.jare.2021.11.009 PMC9039752 35499046

[15] Chu, Hiutung , Arya Khosravi , Indah P. Kusumawardhani , Alice H. K. Kwon , Anilton C. Vasconcelos , Larissa D. Cunha , Anne E. Mayer , et al. 2016. “Gene‐Microbiota Interactions Contribute to the Pathogenesis of Inflammatory Bowel Disease.” Science 352(6289): 1116–1120. 10.1126/science.aad9948 PMC4996125 27230380

[16] Xue, Ming‐Yuan , Hui‐Zeng Sun , Xue‐Hui Wu , Jian‐Xin Liu , and Le Luo Guan . 2020. “Multi‐Omics Reveals That the Rumen Microbiome and Its Metabolome Together With the Host Metabolome Contribute to Individualized Dairy Cow Performance.” Microbiome 8(1): 64. 10.1186/s40168-020-00819-8 PMC7218573 32398126

[17] Kamke, Janine , Sandra Kittelmann , Priya Soni , Yang Li , Michael Tavendale , Siva Ganesh , Peter H. Janssen , et al. 2016. “Rumen Metagenome and Metatranscriptome Analyses of Low Methane Yield Sheep Reveals a Sharpea‐Enriched Microbiome Characterised by Lactic Acid Formation and Utilisation.” Microbiome 4(1): 56. 10.1186/s40168-016-0201-2 PMC5069950 27760570

[18] Li, Fuyong , Thomas C. A. Hitch , Yanhong Chen , Christopher J. Creevey , and Le Luo Guan . 2019. “Comparative Metagenomic and Metatranscriptomic Analyses Reveal the Breed Effect on the Rumen Microbiome and Its Associations With Feed Efficiency in Beef Cattle.” Microbiome 7(1): 6. 10.1186/s40168-019-0618-5 PMC6332916 30642389

[19] Henry A, Paz , Christopher L. Anderson , Makala J. Muller , Paul J., Kononoff , and Samodha C. Fernando . 2016. “Rumen Bacterial Community Composition in Holstein and Jersey Cows Is Different Under Same Dietary Condition and Is Not Affected by Sampling Method.” Frontiers in Microbiology 7: 01206. 10.3389/fmicb.2016.01206 PMC4971436 27536291

[20] Zhang, Meng , Chengbo Liang , Bin Li , Feng Jiang , Pengfei Song , Haifeng Gu , Hongmei Gao , Zhenyuan Cai , and Tongzuo Zhang . 2025. “Gut Microbiome and Diet Contribute to Ecological Niche Differentiation Between Argali (Ovis Ammon Hodgsoni) and Blue Sheep (Pseudois Nayaur) on the Qinghai‐Tibet Plateau.” Communications Biology 8(1): 930. 10.1038/s42003-025-08185-7 PMC12170824 40523923

[21] Zhao, Hongwen , Longjie Sun , Jiali Liu , Bin Shi , Yaopeng Zhang , Ci‐Ren Qu‐Zong , Tsechoe Dorji , et al. 2024. “Meta‐Analysis Identifying Gut Microbial Biomarkers of Qinghai‐Tibet Plateau Populations and the Functionality of Microbiota‐Derived Butyrate in High‐Altitude Adaptation.” Gut Microbes 16(1): 2350151. 10.1080/19490976.2024.2350151 PMC11086029 38715346

[22] Xin, Jinwei , Zhixin Chai , Chengfu Zhang , Qiang Zhang , Yong Zhu , Hanwen Cao , Jincheng Zhong , and Qiumei Ji . 2019. “Comparing the Microbial Community in Four Stomach of Dairy Cattle, Yellow Cattle and Three Yak Herds in Qinghai‐Tibetan Plateau.” Frontiers in Microbiology 10: 1547. 10.3389/fmicb.2019.01547 PMC6636666 31354656

[23] Shabat, Sheerli Kruger Ben , Goor Sasson , Adi Doron‐Faigenboim , Thomer Durman , Shamay Yaacoby , Margret E. , Berg Miller , Bryan A. White , Naama Shterzer , and Itzhak Mizrahi . 2016. “Specific Microbiome‐Dependent Mechanisms Underlie the Energy Harvest Efficiency of Ruminants.” The ISME Journal 10(12): 2958–2972. 10.1038/ismej.2016.62 PMC5148187 27152936

[24] Neu, Alexander T. , Eric E. Allen , and Kaustuv Roy . 2021. “Defining and Quantifying the Core Microbiome: Challenges and Prospects.” Proceedings of the National Academy of Sciences 118(51): e2104429118. 10.1073/pnas.2104429118 PMC8713806 34862327

[25] Wang, Dangdang , Luyu Chen , Guangfu Tang , Junjian Yu , Jie Chen , Zongjun Li , Yangchun Cao , et al. 2023. “Multi‐Omics Revealed the Long‐Term Effect of Ruminal Keystone Bacteria and the Microbial Metabolome on Lactation Performance in Adult Dairy Goats.” Microbiome 11(1): 215. 10.1186/s40168-023-01652-5 PMC10540338 37773207

[26] Qi, Keke , Xiaoming Men , Jie Wu , Bo Deng , and Ziwei Xu . 2022. “Effects of Growth Stage and Rearing Pattern on Pig Gut Microbiota.” Current Microbiology 79(5): 136. 10.1007/s00284-022-02828-2 35303185

[27] Wardman, Jacob F. , Rajneesh K. Bains , Peter Rahfeld , and Stephen G. Withers . 2022. “Carbohydrate‐Active Enzymes (CAZymes) in the Gut Microbiome.” Nature Reviews Microbiology 20(9): 542–556. 10.1038/s41579-022-00712-1 35347288

[28] Gharechahi, Javad , Sajjad Sarikhan , Jian‐Lin Han , Xue‐Zhi Ding , and Ghasem Hosseini Salekdeh . 2022. “Functional and Phylogenetic Analyses of Camel Rumen Microbiota Associated With Different Lignocellulosic Substrates.” npj Biofilms and Microbiomes 8(1): 46. 10.1038/s41522-022-00309-9 PMC9177762 35676509

[29] Gharechahi, Javad , Mohammad Farhad Vahidi , Xue‐Zhi Ding , Jian‐Lin Han , and Ghasem Hosseini Salekdeh . 2020. “Temporal Changes in Microbial Communities Attached to Forages With Different Lignocellulosic Compositions in Cattle Rumen.” FEMS Microbiology Ecology 96(6): fiaa069. 10.1093/femsec/fiaa069 32304321

[30] Guo, Jingling , Runzhou Zhao , Kun Li , Yafang Tan , Likun Wang , Hui Ling , Huan Zhang , et al. 2025. “Altitude Adaptation: The Unseen Work of Gut Microbiota.” hLife 3(1): 5–20. 10.1016/j.hlife.2024.11.004 PMC13681590 42903972

[31] Cumming, Bridgette M. , and Adrie J. C. Steyn . 2015. “Metabolic Plasticity of Central Carbon Metabolism Protects Mycobacteria.” Proceedings of the National Academy of Sciences 112(43): 13135–13136. 10.1073/pnas.1518171112 PMC4629367 26483480

[32] Noor, Elad , Eran Eden , Ron Milo , and Uri Alon . 2010. “Central Carbon Metabolism as a Minimal Biochemical Walk Between Precursors for Biomass and Energy.” Molecular Cell 39(5): 809–820. 10.1016/j.molcel.2010.08.031 20832731

[33] TeSlaa, Tara , Markus Ralser , Jing Fan , and Joshua D. Rabinowitz . 2023. “The Pentose Phosphate Pathway in Health and Disease.” Nature Metabolism 5(8): 1275–1289. 10.1038/s42255-023-00863-2 PMC11251397 37612403

[34] Midha, Ayush D. , Yuyin Zhou , Bruno B. Queliconi , Alec M. Barrios , Augustinus G. Haribowo , Brandon T. L. Chew , Cyril O. Y. Fong , et al. 2023. “Organ‐Specific Fuel Rewiring in Acute and Chronic Hypoxia Redistributes Glucose and Fatty Acid Metabolism.” Cell Metabolism 35(3): 504–516.e5. 10.1016/j.cmet.2023.02.007 PMC10077660 36889284

[35] Zhao, Congcong , Lamei Wang , Shanlin Ke , Xinhua Chen , Ákos Kenéz , Wei Xu , Dangdang Wang , et al. 2022. “Yak Rumen Microbiome Elevates Fiber Degradation Ability and Alters Rumen Fermentation Pattern to Increase Feed Efficiency.” Animal Nutrition 11: 201–214. 10.1016/j.aninu.2022.07.014 PMC9556794 36263411

[36] Brosh, A. , Y. Aharoni , E. Shargal , B. Sharir , M. Gutman , and I. Choshniak . 2004. “Energy Balance of Grazing Beef Cattle in Mediterranean Pasture, the Effects of Stocking Rate and Season.” Livestock Production Science 90(2–3): 101–115. 10.1016/j.livprodsci.2004.03.008

[37] Wang, Weiwei , Yuntao Dong , Wei Guo , Xiao Zhang , A. Allan Degen , Sisi Bi , Luming Ding , Xiang Chen , and Ruijun Long . 2024. “Linkages Between Rumen Microbiome, Host, and Environment in Yaks, and Their Implications for Understanding Animal Production and Management.” Frontiers in Microbiology 15: 1301258. 10.3389/fmicb.2024.1301258 PMC10860762 38348184

[38] Zhang, Jun , Haitao Shi , Yajing Wang , Shengli Li , Zhijun Cao , Shoukun Ji , Yuan He , and Hongtao Zhang . 2017. “Effect of Dietary Forage to Concentrate Ratios on Dynamic Profile Changes and Interactions of Ruminal Microbiota and Metabolites in Holstein Heifers.” Frontiers in Microbiology 8: 2206. 10.3389/fmicb.2017.02206 PMC5684179 29170660

[39] Ding, X. Z. , R. J. Long , M. Kreuzer , J. D. Mi , and B. Yang . 2010. “Methane Emissions From Yak (*Bos Grunniens*) Steers Grazing or Kept Indoors and Fed Diets With Varying Forage:Concentrate Ratio During the Cold Season on the Qinghai‐Tibetan Plateau.” Animal Feed Science and Technology 162(3–4): 91–98. 10.1016/j.anifeedsci.2010.09.007

[40] Han, Yang , Xiaoshuang Liu , Qian Jia , Jiayu Xu , Jinlong Shi , Xiang Li , Guotong Xie , Xiaojing Zhao , and Kunlun He . 2024. “Longitudinal Multi‐Omics Analysis Uncovers the Altered Landscape of Gut Microbiota and Plasma Metabolome in Response to High Altitude.” Microbiome 12(1): 70. 10.1186/s40168-024-01781-5 PMC10996103 38581016

[41] Machmüller, Andrea , Carla R. Soliva , and Michael Kreuzer . 2003. “Effect of Coconut Oil and Defaunation Treatment on Methanogenesis in Sheep.” Reproduction Nutrition Development 43(1): 41–55. 10.1051/rnd:2003005 12785449

[42] Na, Sang Weon , and Le Luo Guan . 2022. “Understanding the Role of Rumen Epithelial Host‐Microbe Interactions in Cattle Feed Efficiency.” Animal Nutrition 10: 41–53. 10.1016/j.aninu.2022.04.002 PMC9117530 35647325

[43] Lane, M. A. , R. L. Baldwin , and B. W. Jesse . 2002. “Developmental Changes in Ketogenic Enzyme Gene Expression During Sheep Rumen Development.” Journal of Animal Science 80(6): 1538–1544. 10.2527/2002.8061538x 12078735

[44] Xiang, Ruidong , Victor Hutton Oddy , Alan L. Archibald , Phillip E. Vercoe , and Brian P. Dalrymple . 2016. “Epithelial, Metabolic and Innate Immunity Transcriptomic Signatures Differentiating the Rumen From Other Sheep and Mammalian Gastrointestinal Tract Tissues.” PeerJ 4: e1762. 10.7717/peerj.1762 PMC4793311 26989612

[45] Xue, Ming‐Yuan , Jia‐Jin Wu , Yun‐Yi Xie , Sen‐Lin Zhu , Yi‐Fan Zhong , Jian‐Xin Liu , and Hui‐Zeng Sun . 2022. “Investigation of Fiber Utilization in the Rumen of Dairy Cows Based on Metagenome‐Assembled Genomes and Single‐Cell RNA Sequencing.” Microbiome 10(1): 11. 10.1186/s40168-021-01211-w PMC8772221 35057854

[46] Depommier, Clara , Amandine Everard , Céline Druart , Hubert Plovier , Matthias Van Hul , Sara Vieira‐Silva , Gwen Falony , et al. 2019. “Supplementation with *Akkermansia Muciniphila* in Overweight and Obese Human Volunteers: A Proof‐of‐Concept Exploratory Study.” Nature Medicine 25(7): 1096–1103. 10.1038/s41591-019-0495-2 PMC6699990 31263284

[47] Gu, Yu , Can Liu , Ningning Zheng , Wei Jia , Weidong Zhang , and Houkai Li . 2019. “Metabolic and Gut Microbial Characterization of Obesity‐Prone Mice Under a High‐Fat Diet.” Journal of Proteome Research 18(4): 1703–1714. 10.1021/acs.jproteome.8b00945 30793608

[48] Ridaura, Vanessa K. , Jeremiah J. Faith , Federico E. Rey , Jiye Cheng , Alexis E. Duncan , Andrew L. Kau , Nicholas W. Griffin , et al. 2013. “Gut Microbiota From Twins Discordant for Obesity Modulate Metabolism in Mice.” Science 341(6150): 1241214. 10.1126/science.1241214 PMC3829625 24009397

[49] Xue, Ming‐Yuan , Yun‐Yi Xie , Yifan Zhong , Xiao‐Jiao Ma , Hui‐Zeng Sun , and Jian‐Xin Liu . 2022. “Integrated Meta‐Omics Reveals New Ruminal Microbial Features Associated With Feed Efficiency in Dairy Cattle.” Microbiome 10(1): 32. 10.1186/s40168-022-01228-9 PMC8849036 35172905

[50] Clemmons, Brooke A. , Brynn H. Voy , and Phillip R. Myer . 2019. “Altering the Gut Microbiome of Cattle: Considerations of Host‐Microbiome Interactions for Persistent Microbiome Manipulation.” Microbial Ecology 77(2): 523–536. 10.1007/s00248-018-1234-9 30033500

[51] Li, Yan , Yingkui Yang , Shatuo Chai , Kaiyue Pang , Xun Wang , Linpeng Xu , Zheng Chen , et al. 2023. “Ruminal Fluid Transplantation Accelerates Rumen Microbial Remodeling and Improves Feed Efficiency in Yaks.” Microorganisms 11(8): 1964. 10.3390/microorganisms11081964 PMC10458777 37630524

[52] DePeters, E. J. , and L. W. George . 2014. “Rumen Transfaunation.” Immunology Letters 162(2): 69–76. 10.1016/j.imlet.2014.05.009 25262872

[53] Chen, Shifu , Yanqing Zhou , Yaru Chen , and Jia Gu . 2018. “fastp: An Ultra‐Fast All‐In‐One FASTQ Preprocessor.” Bioinformatics 34(17): i884–i890. 10.1093/bioinformatics/bty560 PMC6129281 30423086

[54] Li, Dinghua , Chi‐Man Liu , Ruibang Luo , Tak‐Wah Lam , and Sadakane . 2015. “MEGAHIT: An Ultra‐Fast Single‐Node Solution for Large and Complex Metagenomics Assembly Via Succinct *de bruijn* Graph.” Bioinformatics 31(10): 1674–1676. 10.1093/bioinformatics/btv033 25609793

[55] Fu, Limin , Beifang Niu , Zhengwei Zhu , Sitao Wu , and Weizhong Li . 2012. “CD‐HIT: Accelerated for Clustering the Next‐Generation Sequencing Data.” Bioinformatics 28(23): 3150–3152. 10.1093/bioinformatics/bts565 PMC3516142 23060610

[56] Li, Ruiqiang , Yingrui Li , Karsten Kristiansen , and Jun Wang . 2008. “SOAP: Short Oligonucleotide Alignment Program.” Bioinformatics 24(5): 713–714. 10.1093/bioinformatics/btn025 18227114

[57] Haas, Brian J. , Alexie Papanicolaou , Moran Yassour , Manfred Grabherr , Philip D. Blood , Joshua Bowden , Matthew Brian Couger , et al. 2013. “De Novo Transcript Sequence Reconstruction From RNA‐seq Using the Trinity Platform for Reference Generation and Analysis.” Nature Protocols 8(8): 1494–1512. 10.1038/nprot.2013.084. PMC3875132 23845962

[58] Buchfink, Benjamin , Chao Xie , and Daniel H. Huson . 2015. “Fast and Sensitive Protein Alignment Using DIAMOND.” Nature Methods 12(1): 59–60. 10.1038/nmeth.3176 25402007

[59] Benjamini, Yoav , and Yosef Hochberg . 1995. “Controlling the False Discovery Rate: A Practical and Powerful Approach to Multiple Testing.” Journal of the Royal Statistical Society Series B: Statistical Methodology 57(1): 289–300. 10.1111/j.2517-6161.1995.tb02031.x

[60] Butler, Andrew , Paul Hoffman , Peter Smibert , Efthymia Papalexi , and Rahul Satija . 2018. “Integrating Single‐Cell Transcriptomic Data Across Different Conditions, Technologies, and Species.” Nature Biotechnology 36(5): 411–420. 10.1038/nbt.4096 PMC6700744 29608179

