# Supplementary material for: The role of the gut microbiome in the regulation of high‐altitude adaptation
Source: Imeta. 2026 Jan 19;5(1):e70104. doi: 10.1002/imt2.70104 (PMC13726630; doi:10.1002/imt2.70104)
Supplement: Supplementary file 1 [file IMT2-5-e70104-s001.docx]

**Supplementary Information to**

**Mechanisms of the gastrointestinal microbiome in the regulation of high-altitude adaptation: Experimental evidence from the long-term adapted yak (*Bos grunniens*)**

Xinyu Zhang^1^ | Senlin Zhu^2, 3^ | Michael Kreuzer^2^ | Shoukun Ji^4^ | Wei Wang^1^ | Yanliang Bi^5*^ | Shengli Li^1*^

^1^State Key Laboratory of Animal Nutrition and Feeding, College of Animal Science and Technology, China Agricultural University, 100194, Beijing, China

^2^ETH Zurich, Animal Nutrition, Institute of Agricultural Sciences, Department of Environmental Systems Science, ETH Zurich, Eschikon 27, CH-8315 Lindau, Switzerland

^3^Laboratory of Dairy Cow Genetic Improvement and Milk Quality Research of Zhejiang Province, College of Animal Sciences, Zhejiang University, Hangzhou 310058, China

^4^College of Animal Science and Technology, Hebei Agricultural University, Baoding 071000, China

^5^Key Laboratory of Feed Biotechnology of the Ministry of Agriculture and Rural Affairs, Institute of Feed Research of Chinese Academy of Agricultural Sciences, Beijing 100193, China

**Correspondence:**

Shengli Li

State Key Laboratory of Animal Nutrition and Feeding

College of Animal Science and Technology, China Agricultural University, 100194, Beijing, China.

Email: [lisheng0677@163.com](mailto:lisheng0677@163.com)

Yanliang Bi

Key Laboratory of Feed Biotechnology of the Ministry of Agriculture and Rural Affairs, Institute of Feed Research of Chinese Academy of Agricultural Sciences, Beijing 100193, China

Email: [biyanliang@caas.cn](mailto:biyanliang@caas.cn)

**Figure S1:** Differences between Holstein cows (H) and yaks (Y) in alpha diversity based on Wilcoxon rank sum test in rumen microbiome by metagenomic analysis (n = 20 per group; Experiment 1). All microbial species (A), archaea (B), and bacteria (C).

**Figure S2:** Differences between Holstein cows (H) and yaks (Y) in rumen microbial composition and its association with end products of ruminal fermentation, based on metagenomic analysis (n = 20 per group; Experiment 1). (A) Pie chart of unique microbe species and groups of taxa of archaea and bacteria for Holstein cows and yaks. (B) Differences in common archaeal species composition between Holstein cows and yaks determined by Linear discriminant analysis Effect Size. Heatmap of correlations between rumen-specific archaea (C for Holstein cows, D for yak group; |*r*| > 0.6) as well as bacteria (E for Holstein cows, F for yak group; |*r*| > 0.7) and end products of ruminal fermentation in Holstein cows and yaks, Heatmap of correlation between the abundance of individual archaeal species common to Holstein cows and yaks and end products of ruminal fermentation in Holstein cows (G) and yaks (H). Heatmap of correlations between the abundance and individual bacterial species common to Holstein cows (I) and yaks (J) and end products of ruminal fermentation. A/P: acetate-to-propionate ratio.

**Figure S3:** Differences in CAZy and KEGG functional profiles between Holstein cows (H) and yaks (Y), based on metagenomic analysis (n = 20 per group; Experiment 1). Pie charts of CAZyme composition at the class and family levels for Holstein cows (A) and yaks (B). Functional differences of CAZyme at the class level (C) and family level (D) between Holstein cows and yaks with Wilcoxon rank sum test. (E) Carbohydrate degradation enzyme function differences with Wilcoxon rank sum test. (F-G) Fold changes of Kyoto Encyclopedia of Genes and Genomes (KEGG) pathways identified in the metagenomes. Pathways were sorted for amino acid metabolism (F), and lipid metabolism (G); The Wilcoxon rank‐sum test was used for mean comparison. * adjusted *p*  <  0.05; ** adjusted *p*  <  0.01; *** adjusted *p* <  0.001.

**Figure S4:** Chart showing the established mediating effect models.

**Figure S5:** Differences between the Holstein cows (H) and the yaks (Y) in rumen microbial composition and function in the rumen fluid as determined by metatranscriptome analysis (n = 10 per group; Experiment 1). Differences between Holstein cows and yaks in alpha diversity of all microbial species (A) archaea (B) and bacteria (C) in rumen microbiome. (D) Pie chart of unique rumen microbe species and groups of taxa of archaea and bacteria for Holstein cows and yaks. Differences in common archaeal species composition (E) between Holstein cows and yaks determined by Linear discriminant analysis Effect Size. (F) Carbohydrate degradation enzyme function differences with Wilcoxon rank sum test. (G, H) Fold changes of KEGG pathways identified in the metagenomes. Pathways were sorted for amino acid metabolism (G), and lipid metabolism (H); The Wilcoxon rank‐sum test was used for mean comparison. * adjusted *p*  <  0.05; ** adjusted *p*  <  0.01; *** adjusted *p*  <  0.001.

**Figure S6:** Differences between the Holstein cows (H) and the yaks (Y) as illustrated by a rumen epithelial single cell map (n = 3 per group; Experiment 1). (A) Distribution of cells in each sample. (B) Distribution of cells in each cell clustering. Marker gene expression in single cell type of Holstein cows (C)and yaks (D). Uniform manifold approximation and projection maps of cell types from Holstein cows (E) and yaks (F) rumen samples, with each cell color indicating the associated cell subtypes. Sn = single-nucleus RNA sequencing.

**Figure S7:** Cell subtype correlations and functional differences between Holstein cows (H) and the yaks (Y) (n = 3 per species; Experiment 1). (A) Heat map of the similarities between Holstein cows and yaks in cell subtypes, where the redder the color the stronger the correlation. (B) Differences between Holstein cows and yaks in the top 15 gene ontology function pathways. (C-E) scores of different cell subtypes from groups Holstein cows and yaks in the VFA catabolic process, ketone body biosynthesis metabolism, and fatty acid beta-oxidation. Sn = single-nucleus RNA sequencing. E = epithelial cell.

**Figure S8:** Effect of yak rumen fluid transplantation on microbiome composition and functional profiles in Holstein cows (FR= Fresh rumen fluid group) at 6 h, and on Day 7 and Day 28 post transplantation. (n = 10 per group; Experiment 2) (A) Key differential genera at 6 h, and on Day 7 and Day 28 after rumen fluid transplantation. (B) Overview of module differences (*p* < 0.05) between Holstein cows (H) and yaks (Y) in metagenomic and metatranscriptomic profiles, alongside alterations in carbon metabolism modules following rumen fluid transplantation as determined the using Kruskal-Wallis test. (C) Differences in carbon metabolism modules following rumen fluid transplantation based on Wilcoxon rank sum test.

**Correlations between the abundance of individual rumen microbial species determined by metagenomics analysis and being either unique to Holstein cows or yaks or common to both animal species and ruminal fermentation traits**

Among the archaeal species unique to Holstein cows (Figure S2C), the relative abundance of *Natroncoccus pandeyae*, *Methanolobus zinderi*, and *Archaeon GW2011 AR20* in the archaea was positively correlated with the concentrations of acetic acid, propionic acid, butyric acid, valeric acid and total volatile acids (VFA), respectively (*p <* 0.05, |*r*| > 0.6), while *Archaeon GW2011 AR20* was negatively correlated with the acetic acid-to-propionic acid ratio (A/P ratio; *p <* 0.05, |*r*| > 0.6), and *Methanolobus zinderi* was negatively correlated with ammonia nitrogen (NH_3_-N) (*p <* 0.05, |*r*| > 0.6).

Among the archaeal species unique to yak (Figure S2D), the relative abundance of *Methanospirillum hungatei* in Archaea was negatively correlated with acetic acid (*p <* 0.05, |*r*| > 0.6), and *Candidatus Woesearchaeota archaeon CG11* was negatively correlated with the A/P ratio (*p <* 0.05, |*r*| > 0.6).

As for bacterial species unique to Holstein cows (Figure S2E), the relative abundance of *Flavobacterium sharifuzzamanii* was positively correlated with the concentrations of acetic acid, butyric acid and total VFA (*p <* 0.05, |*r*| > 0.7), *Kamptonema sp PCC 6506* was positively correlated with the concentration of valeric acid (*p <* 0.05, |*r*| > 0.7); *Puniceicoccaceae bacterium K14*, *Sediminbacillus halophilus*, *Massilia sp.* were positively correlated with pH (*p <* 0.05, |*r*| > 0.7); *Parcubacteria group bacterium CG1 02 44 31* and *Legionella clemsonensis* were positively correlated with NH_3_-N (*p <* 0.05, |*r*| > 0.7); *Photorhabdus uminescens* was negatively correlated with the concentrations of acetic acid and total VFA (*p <* 0.05, |*r*| > 0.7); propionic acid was correlated with *Lactobacillus sp.* *PV034* and *Devosia sp. JXJ CY 41* (*p <* 0.05, |*r*| > 0.7); butyric acid was negatively correlated with *Bacillus dafuensis* (*p <* 0.05, |*r*| > 0.7); valeric acid was negatively correlated with *Metabacillus endolithicus*, *Devosia sp. JXJ CY 41* and *Acidobacteria bacterium RIFCSPLOWO2 02 FULL 61 28* (*p <* 0.05, |*r*| > 0.7); pH value was negatively correlated with *Lentisphaerae bacterium RIFOXYA12 64 32*, *Halomonas xianhensis*, *Caldicellulosiruptor kronotskyensis*, and *Acinetobacter guillouiae* (*p <* 0.05, |*r*| > 0.7); NH_3_-N was negatively correlated with *Pedobacter kyungheensis* (*p <* 0.05, |*r*| > 0.7).

In terms of bacterial species unique to yak (Figure S2F), acetic acid was positively correlated with *Blautia sp.TF12-12AT* and *Pseudomonas viciae* (*p <* 0.05, |*r*| > 0.7), but negatively correlated with *Oscillibacter sp.57_20* (*p* < 0.05, |*r*| > 0.7); propionic acid was negatively correlated with *Lapidilactobacillus mulanensis* (*p <* 0.05, |*r*| > 0.7), but positively correlated with *Enterococcus sp S22* (*p <* 0.05, |*r*| > 0.7); isopropionic acid was negatively correlated with *Pseudodesulfovibrio sp. JC047*, *Pedobacter puniceum*, and *lgnavibacterium bacterium_GWA2_55_25* (*p <* 0.05, |*r*| > 0.7); isovaleric acid was negatively correlated with *Pedobacter puniceum* (*p <* 0.05, |*r*| > 0.7); the A/P ratio was negatively correlated with *Enterococcus sp S22*, *Bacillus sp. 6YEL31* and *Bacillus pacificus* (*p <* 0.05, |*r*| > 0.7); pH was positively correlated with *Cyanobium sp. 49614 E6* (*p <* 0.05, |*r*| > 0.7); NH_3_-N was negatively correlated with *Thiopseudomonas sp.* (*p <* 0.05, |*r*| > 0.7); and was positively correlated with *Dictyobacter vulcani* and *Aminivibro sp.* (*p <* 0.05, |*r*| > 0.7).

Concerning the archaeal species shared between Holstein cows and yak, in Holstein cows rumen fluid blood urea nitrogen (BUN) was positively correlated with *Methanosphaera sp. rholeuAM6*, *Methanosphaera sp. rholeuAM270*, *Methanosphaera sp.* and *Methanocaldococcus villosus*; pH was positively correlated with *Bathyarchaeota archaeon A05DMB−2* and negatively correlated with *ANME−2 cluster archaeon*; valeric acid was negatively correlated with *Pacearchaeota archaeon* (all *p <* 0.05, |*r*| > 0.5, Figure S2G).

In yak rumen fluid, *Methanoperedenaceae archaeon GB37* was positively correlated with propionic acid and butyric acid concentrations and negatively correlated with A/P ratio and NH_3_-N; *Methanobacteriales archaeon HGW−Methanobacteriales−2* was positively correlated with total VFA concentrations (all *p <* 0.05, |*r*| > 0.5, Figure S2H).

Concerning the bacterial species shared in Holstein cows and yak, the abundance of 21 species belonging to the genus *Prevotellaceae* in the rumen fluid of Holstein cows were positively correlated with pH (*p <* 0.05, |*r*| > 0.7, Figure S2I). *Treponema sp.*, *Prevotellaceae bacterium MN60*, *Prevotellaceae bacterium HUN15*, and *Prevotellaceae bacterium* were also negatively correlated with the A/P ratio (*p <* 0.05, |*r*| > 0.7); 11 species belonging to the genus *Prevotellaceae* were negatively correlated with BUN (*p <* 0.05, |*r*| > 0.7), *SR1 bacterium human oral taxon HOT−345*, *Gracilibacteria bacterium*, *Fibrobacter sp. UWB2*, *Fibrobacter succinogenes* and three *Prevotellaceae* families (*Prevotellaceae bacterium MN60*, *Prevotellaceae bacterium HUN156*, *Prevotellaceae bacterium*) were negatively correlated (*p <* 0.05, |*r*| > 0.7; *Ruminococcus sp. M6* was negatively correlated with the concentrations of acetic acid, butyric acid, isobutyric acid, isovaleric acid and total VFA (*p <* 0.05, |*r*| > 0.7); *Ruminococcaceae bacterium P7* was negatively correlated with the concentrations of acetic acid, butyric acid and total VFA (*p <* 0.05, |*r*| > 0.7); the concentrations of acetic acid, butyric acid and total VFA were positively correlated with *Bacteroidaceae bacterium* and *bacteria P201* (*p <* 0.05, |*r*| > 0.7). In addition, the total VFA concentration was also correlated with *Prevotellaceae bacterium MN60* (*p <* 0.05, |*r*| > 0.7).

In the rumen fluid of yak (Figure S2J), NH_3_-N was negatively correlated with *Mycoplasma sp.* (*p <* 0.05, |*r*| > 0.7); isovaleric acid was negatively correlated with *Mogibacterium sp.*, *Coriobacteriaceae bacterium*, *Coprococcus catus* and *Clostridiales bacterium FE2011* (*p <* 0.05, |*r*| > 0.7); isobutyric acid was negatively correlated with *Bacte roidetes bacterium* and *Bacteroidales bacterium* were negatively correlated (*p <* 0.05, |*r*| > 0.7); isobutyric acid and valeric acid were both correlated with *Solobacterium sp*., *Lachnospiraceae bacterium*, *Eggerthellaceae bacterium*, *Coriobacteriaceae bacterium*, *Coprococcus catus*, *Clostridiales bacterium FE2011*, *Clostridiales bacterium* , *Clostridia bacterium*, *Blautia sp.* and *Anaerolineaceae bacterium* (*p* < 0.05, |*r*| > 0.7); in addition, isobutyric acid was positively correlated with *Mogibacterium sp.* and *Clostridium sp.* (*p <* 0.05, |*r*| > 0.7); valeric acid was negatively correlated with *Bacteroidetes bacterium* (*p <* 0.05, |*r*| > 0.7); butyric acid was positively correlated with *Clostridiales bacterium* (*p* < 0.05, |*r*| > 0.7).
